# *Bergeyella cardium* variant induces a unique cytoplasmic vacuolization cell death floatptosis in macrophage

**DOI:** 10.1038/s41421-025-00840-x

**Published:** 2025-10-21

**Authors:** Rudi Mao, Hongwei Pan, Luyu Yang, Zhenyu Fan, Yanfeng Li, Xinran Yu, Zhen Li, Ying Chen, Yang Yu, Wei Wang, Chengjiang Gao, Jun Peng, Tao Xu, Yi Zhang, Xiaopeng Qi

**Affiliations:** 1 Key Laboratory for Experimental Teratology of the Ministry of Education, Department of Clinical Laboratory/Qilu Hospital, Advanced Medical Research Institute, Cheeloo College of Medicine, Shandong University, Jinan, China; 2 Tumor Research and Therapy Center, Shandong Provincial Hospital Affiliated to Shandong First Medical University, 324 Jing-Wu Road, Jinan, China; 3 Department of Clinical Laboratory, Qilu Hospital of Shandong University, Jinan, China; 4 Shandong Engineering Research Center of Biomarker and Artificial Intelligence Application, Jinan, China; 5 Advanced Medical Research Institute, Cheeloo College of Medicine, Shandong University, Jinan, China; 6 Department of Immunology and Key Laboratory of Infection and Immunity of Shandong Province & Key Laboratory for Experimental Teratology of Ministry of Education, Department of Immunology, School of Basic Medical Sciences, Shandong University, Jinan, China; 7 Department of Hematology, Qilu Hospital, Cheeloo College of Medicine, Shandong University, Jinan, China; 8 State Key Laboratory for Innovation and Transformation of Luobing Theory, Key Laboratory of Cardiovascular Remodeling and Function Research of MOE, NHC, CAMS and Shandong Province, Department of Cardiology, Qilu Hospital of Shandong University, Jinan, China

**Keywords:** Cell death, Innate immunity

## Abstract

Bacterial pathogens have evolved multiple mechanisms to modulate host cell death, evade host immunity, and establish persistent infection. Here, we show that an infective endocarditis causative pathogen, *Bergeyella cardium*, is frequently detected in oral specimens from clinical patients. A variant strain of *Bergeyella cardium* (*BCV*) induces unique cytoplasmic vacuolization cell death and minor apoptosis-like cell death in macrophages. The cytoplasmic vacuolization cell death triggered by *BCV* is characterized by Fused LysosOme-Associated Termination (floatptosis) and is inhibited by the sodium channel inhibitor amiloride. Moreover, outer membrane vesicles (OMVs) or transfection of barrel-like membrane proteins, lipocalin, β-barrel, and PorV, dramatically induce cytoplasmic vacuolization. Endosomal solute carrier family 9 member A9 (SLC9A9) plays important roles in the process of *BCV*-, OMVs-, and barrel-like proteins-triggered cytoplasmic vacuolization cell death via promoting vacuole fusion. SLC9A9 deficiency or amiloride administration increases host defense against *BCV* infection. These findings contribute to developing novel approaches to modulate cytoplasmic vacuolization cell death and treat infectious diseases.

## Introduction

Host cell death is an intrinsic immune response that plays dual roles in host defense against bacterial infection^1^. Bacterial pathogens have evolved strategies to manipulate host cell death and survival pathways, creating an intracellular replication niche and promoting dissemination^2^. The major programmed cell death pathways, including apoptosis, necroptosis, and pyroptosis, have been extensively studied, and many virulence factors of bacterial pathogens can act through these pathways to increase pathogenicity^3–5^. Cytoplasmic vacuolization is a morphological phenomenon characterized by the dilation and fusion of the endoplasmic reticulum (ER) or endosomal–lysosomal organelles, which can be associated with cell death or promote cell survival depending on the properties of chemical and infectious agents^6^. Many bacterial cytotoxins can induce cytoplasmic vacuolization of multiple organelles and cell death in different types of cells. Pathogenic *E. coli* subtilase cytotoxin SubAB induces vacuolization of the intracellular organelles through interaction with plasma membrane α2β1 integrin receptors^7^ and triggers Bak/Bax conformational changes, cytochrome c release and downstream apoptosis^8^. Community-acquired respiratory distress syndrome toxin (CARDS) from *Mycoplasma pneumoniae* recognizes membrane Annexin A2 protein as a functional receptor. It elicits extensive vacuolization in mammalian cells without knowing the nature of vacuoles and cell death activity^9,10^. *Helicobacter pylori’s* vacuolating toxin A (VacA) is the best-studied vacuolating pore-forming toxin (PFT). VacA interacts with multiple host cell surface proteins for membrane anion-selective channel formation, internalization, and vacuolization of both early and late endosomes with the activation of v-ATPase proton pump, leading to osmotic swelling of endosomes^11–13^. Meanwhile, many studies revealed that VacA not only induced the activation of proapoptotic proteins Bax/Bak and mitochondrial fragmentation for apoptotic cell death, but also triggered programmed necrosis via PARP activation independent of vacuolization, indicating vacuolization is not the direct cause for cell death^14–16^. Similar to VacA, *Vibrio cholerae* toxin VCC forms an anion-selective channel in cells and causes extensive vacuolization of late endosomes and of the trans-Golgi network through autophagy, but VCC-induced vacuolization is not the cause of cell death^17,18^. Instead, the vacuolated organelles, in some cases, can accumulate bacterial toxins to reduce stress and increase the cell survival potential. The relationship between vacuolization and cell death remains unclear, despite the extensive data available on bacterial toxins that induce cytoplasmic vacuolization and cell death. It is still unknown whether the vacuolization phenomenon is a programmed cell response^6^.

Three types of nonapoptotic cytoplasmic vacuolization cell death have been defined, including paraptosis characterized by extensive cytoplasmic vacuolation associated with the dilation of ER/mitochondria^19^, triaptosis distinguished by early endosome vacuolization^20^, and methuosis marked by the vacuolization of late endosome and lysosome^21^. Methuosis is the best-studied type of cytoplasmic vacuolization death in tumor cells and was initially described as active Ras-triggered cell death^21,22^. Recently, increasing evidence has indicated that multiple small-molecule compounds can induce methuosis and suppress cell growth, such as chalcone-related small molecules, MIPP, MOMIPP, epimedokoreanin C, and azaindole-based compounds, which have therapeutic potential in cancer treatment^23–25^. However, the role of cytoplasmic vacuolization in the immune cells for the inflammatory response and immune evasion has not been investigated.

We previously isolated an emerging pathogen strain with a flat dry and rough morphotype colony, *Bergeyella cardium* (*BC*), from a patient with infective endocarditis^26^. In the present study, we found that *BC*-unique sequences were frequently detected in oral microbiomics from clinical patients. We observed that a variant strain of *BC* (*BCV*) had a smooth morphotype and increased resistance to serum complement-dependent clearance. Whole-genome sequencing revealed high sequence identity between *BC* and *BCV*. Notably, *BCV* infection triggered robust cytoplasmic vacuolization cell death and minor apoptosis-like cell death in different cells. The vacuoles induced by *BCV* were colocalized with the late endosomal and lysosomal markers Rab7 and LAMP1. OMVs derived from *BCV* and transfection of lipocalin, β-barrel, and PorV of *BCV* both induced the formation of cytoplasmic vacuolization cell death. SLC9A9 was important for the cytoplasmic vacuolization cell death induced by *BCV* through promoting vacuole fusion. The intracellular propagation and in vivo pathogenicity of *BCV* were significantly more potent than those of *BC*, and SLC9A9 deficiency or amiloride administration increased host defense against *BCV* infection. Our work offers novel mechanistic insights into cytoplasmic vacuolization cell death mechanisms and provides potential therapeutic targets for treating infectious diseases.

## Results

### *BCV* is prone to high pathogenicity

**Fig. 1:**
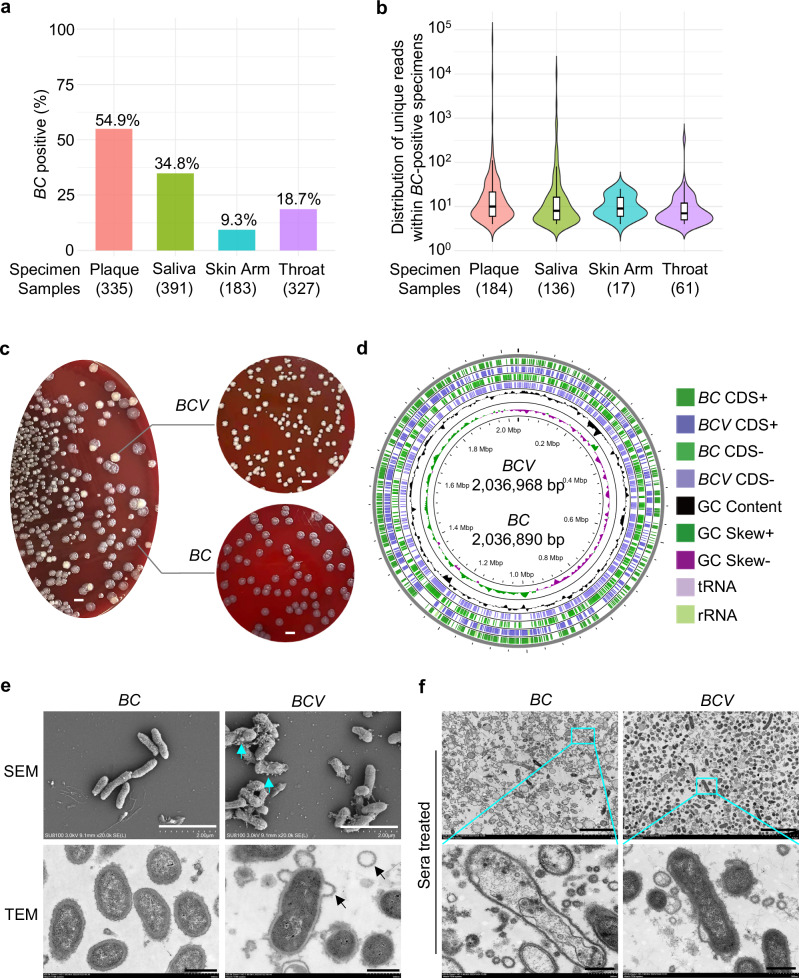
Characterization of *BC* and *BCV.* **a** The prevalence of *BC* in clinical patients. *BC*-positive is defined as *BC*-unique reads (150 nt in length) ≥ 3. The numbers below each bar indicate the corresponding numbers of patients. **b** Distribution of *BC*-unique reads within *BC*-positive specimens. The numbers below each bar indicate the corresponding numbers of *BC*-positive patients. **c** Bacterial colony morphology of *BC* and *BCV*. *BC* and *BCV* were cultured on Columbia blood agar plates for 96 h. Scale bars, 3 mm. **d** Circular genome map of *BCV* with its alignment with *BC*. The circular genome map includes the following, from outer to inner rings: *BC* CDS+, coding sequences (CDS) on the forward strand of the *BC* genome; *BCV* CDS+, CDS on the forward strand of the *BCV* genome; *BC* CDS–, CDS on the reverse strand of the *BC* genome; *BCV* CDS–, CDS on the reverse strand of the *BCV* genome; GC content, which helps identify genomic islands and horizontal gene transfer events; GC skew, indicating the over- or under-abundance of G or C between the leading and lagging DNA strands, which is often used to identify the origin and terminus of replication; and the locations of tRNAs and rRNAs in the genome. The *BC* genome is 2,036,890 bp long, and the *BCV* genome is 2,036,968 bp long. **e** SEM and TEM analysis of *BC* and *BCV*. The arrows indicate puff-up and blebbing of *BCV*. Scale bars, 2 μm for SEM and 500 nm for TEM. **f** TEM analysis of *BC* and *BCV* bacteria treated with human sera for 2 h. Sera-treated *BC* and *BCV* were precipitated, fixed, and sectioned for TEM analysis. Scale bars, 5 μm for upper and 500 nm for lower. Data are representative of 3 independent experiments with similar results (**c**, **e**, **f**).

To analyze the prevalence of *BC* in clinical patients, which was previously isolated from a patient with infective endocarditis^26^, we detected *BC*-unique sequences using the resource data of pan-body pan-disease microbiomics^27^. Notably, *BC* was frequently detected in plaque (54.9% in 335 specimens), saliva (34.8% in 391 specimens), skin arm (9.3% in 183 specimens), and throat (18.7% in 327 specimens) from clinical patients (Fig. 1a, b; Supplementary Table S1). The high proportion of *BC*-positive specimens in clinical patients, particularly oral specimens, underscores the certain noncausal infectious diseases potentially caused by *BC*. To further investigate the detailed bacterial characteristics and pathogenesis of *BC*, we spread the bacterial stock on Columbia blood agar plates. Interestingly, we found the colonies exhibited two distinct colony morphologies (Fig. 1c). The *BC* colonies were flat, dry, and rough. In contrast, the *BCV* colonies were raised and smooth (Fig. 1c). We performed whole-genome sequencing of *BC* and *BCV* colonies, and the circular genome lengths of *BC* and *BCV* were 2,036,890 bp and 2,036,968 bp, respectively (Fig. 1d; GenBank CP029149 and CP114055). The genomic homology between *BC* and *BCV* was approximately 99.99%. We compared the *BC* and *BCV* genomes and confirmed that the *BCV* genome harbored three insertions located in the noncoding region, the *PorV* gene, and a hypothetical gene of the *BC* genome (Supplementary Fig. S1a–f). The 24-bp insertion in the *PorV* gene caused an 8-amino-acid insertion in the PorV protein of *BCV*, resulting in a PorV protein longer than that of *BC* (Supplementary Fig. S1c, d). The 13-bp insertion in the hypothetical gene of *BC* caused a frameshift mutation, which produced a new BatD family protein in *BCV* (Supplementary Fig. S1e, f). PorV is a shuttle protein in the outer membrane of the type IX secretion system (T9SS) that mediates the secretion of major virulence factors^28^. To investigate whether the insertion of 8 amino acids in PorV of *BCV* (residues 344–351) led to its structural difference from *BC*, we performed structure prediction analysis using trRosseta^29^. According to the predicted structure, *BCV* PorV folded into a 14-strand β-barrel, and residues 344–351 folded into the β15 of the β-barrel (Supplementary Fig. S1g). *BC* PorV was also predicted to be a 14-strand β-barrel (Supplementary Fig. S1h). However, the predicted structure of *BC* PorV was less reliable in the β14–β15 region, as shown by the lower LDDT in this region (Supplementary Fig. S1g, h). Therefore, compared with *BCV* PorV, deletion of 8 amino acids in *BC* PorV caused structural changes in PorV, which might affect the secretion of virulence factors through interactions with binding partners.

We performed electron microscopy analysis to characterize the phenotypes of *BC* and *BCV* in detail. Scanning electron microscopy (SEM) and transmission electron microscopy (TEM) revealed that *BCV* bacteria had multiple membrane puff-ups and blebbing (Fig. 1e). Serum resistance of bacteria is essential for their persistence in the bloodstream to cause infection and for the development of antibiotic tolerance, which is recognized as a stepping stone toward antibiotic resistance^30–32^. Notably, the capacity for serum resistance in *BCV* was much greater than in *BC* (Fig. 1f; Supplementary Fig. S2a), whereas antibiotic resistance was comparable between *BC* and *BCV* (Supplementary Fig. S2b). To define the genes differentially expressed between *BC* and *BCV*, we performed RNA sequencing (RNA-seq) analysis of *BC* and *BCV* cultured on Columbia blood agar plates for 96 h. Overall, 89 and 487 genes were upregulated and downregulated, respectively, in *BCV* (Supplementary Fig. S2c and Table S2). Notably, the genes involved in bacterial invasion and colonization, stress adaptation, and secretion systems, including types I and IX, were highly expressed in *BCV* (Supplementary Fig. S2d, e). Conversely, the genes that encode proteins involved in immunogenicity and pathogenicity, such as lipopolysaccharide (LPS) and lipoteichoic acid (LTA) biosynthesis, were dramatically downregulated in *BCV* (Supplementary Fig. S2f, g). These results indicate that *BC* is highly prevalent in clinical patients, and *BCV* might be a more virulent *BC* variant strain.

### *BCV* induces lysosomal fusion-mediated cytoplasmic vacuolization cell death

**Fig. 2:**
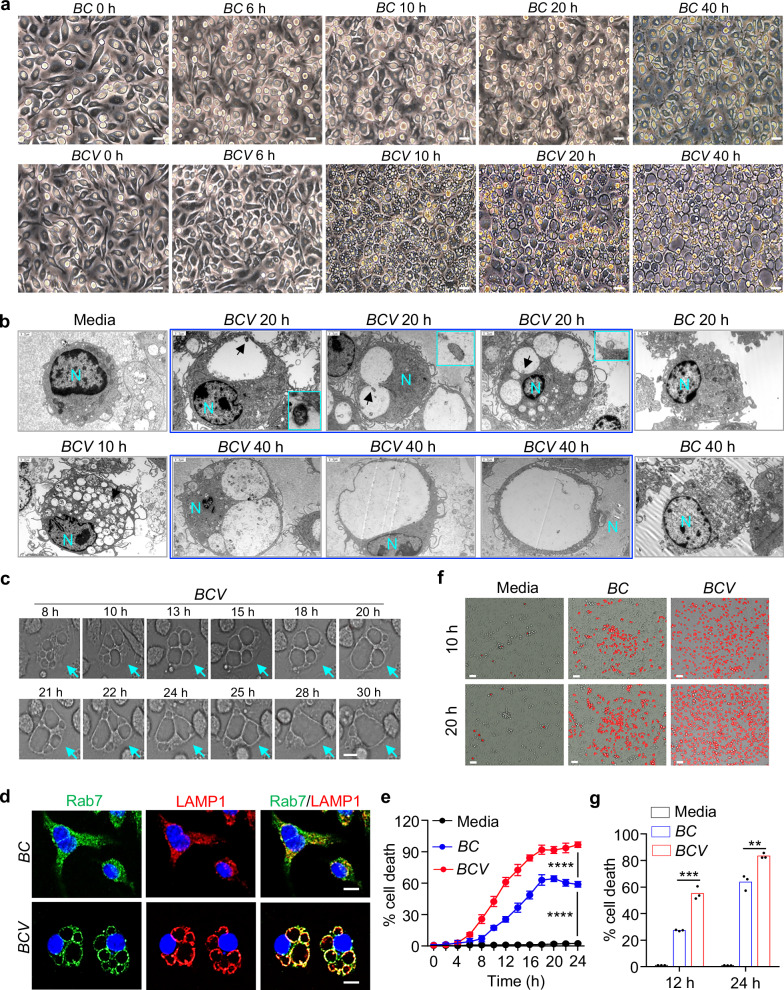
*BCV* infection triggers cytoplasmic vacuolization cell death. **a** Microscopic analysis of WT BMDMs infected with *BC* or *BCV* (400 MOI) for the indicated time. Scale bars, 30 μm. **b** TEM analysis of WT BMDMs infected with *BC* or *BCV* (400 MOI) for the indicated time. N indicates the cell nucleus; arrows indicate the intracellular *BCV* and enlarged images are shown. Scale bars, 1.2 μm. **c** Live-cell imaging of WT BMDMs infected with *BCV* (200 MOI) for the indicated time. Scale bar, 10 μm. **d** Confocal microscopy analysis of Rab7 and LAMP1 in *BC*- and *BCV*-infected (400 MOI) WT BMDMs for 20 h. Scale bars, 20 μm. **e** Real-time quantitative live-cell imaging and analysis of cell death in uninfected WT BMDMs and WT BMDMs infected with *BC* or *BCV* (400 MOI) (*n* = 16 random fields; 3 independent experiments). **f** Representative images of PI staining of uninfected BMDMs and BMDMs infected with *BC* or *BCV* (400 MOI) for the indicated time, as shown in (**e**). Scale bars, 20 μm. **g** LDH analysis of WT BMDMs infected with *BC* or *BCV* (400 MOI) for the indicated time (*n* = 3 biologically independent samples). Data are from 3 independent experiments (**g**) or representative of 3 independent experiments with similar results (**a**–**f**). For (**g**), data represent Mean ± SEM, and two-sided Student’s *t*-test without multiple-comparisons correction was used. For (**e**), two-way ANOVA was used. **P < 0.01, ***P < 0.001, ****P < 0.0001.

To investigate the host cell death response triggered by *BC* and *BCV* infection, we treated bone marrow-derived macrophages (BMDMs) with *BC* or *BCV* for different durations. Notably, *BCV* infection triggered cytoplasmic vacuolization beginning around 6 h post-infection, significant cytoplasmic vacuolization at 10 h post-infection, and large vacuoles and single vacuole-occupied cells after 20 h post-infection (Fig. 2a; Supplementary Fig. S3a). However, this phenomenon was not observed in *BC*-infected BMDMs (Fig. 2a). In addition to BMDMs, *BCV* infection also triggered cytoplasmic vacuolation in L929 cells, immortalized BMDMs (iBMDMs), and the human cardiomyocyte cell line AC16 (Supplementary Fig. S3b–d). To closely examine vacuolization, we performed TEM analysis of *BC*- and *BCV*-infected BMDMs. Notably, *BCV* infection caused the formation of multiple single membrane-bound vacuoles at 10 h post-infection (Fig. 2b). The size of the vacuoles was enlarged, and the number was reduced due to the fusion of vacuoles during the later phase of infection (Fig. 2b; Supplementary Fig. S3e). The process of vacuole fusion and enlargement was also confirmed by live-cell imaging analysis, and plasma membrane permeabilization indicated by Propidium iodide (PI) staining occurred at a later time of the vacuolization process (Fig. 2c; Supplementary Video S1). The vacuoles triggered by *BCV* were labeled with LAMP1 and Rab7 but not the early endosome markers Rab5 or EEA1 (Fig. 2d; Supplementary Fig. S3f–h), which indicated that the vacuoles were derived from lysosomes and late endosomes. In addition, the density of *BCV* infection-induced vacuole-containing cells was lower than that of normal BMDMs, and vacuolated cells were separated at the middle layer of the 40% Percoll gradient (Supplementary Fig. S3i). Cell membrane disruption was observed in *BC*-infected BMDMs at later time points (Fig. 2b). Remarkably, the cell death caused by *BCV* infection was much more severe than that caused by *BC* infection, as determined by real-time quantification of cell death and LDH release assay (Fig. 2e–g).

### *BCV*-triggered cytoplasmic vacuolization cell death is distinct from methuosis

Methuosis is recognized as nonapoptotic cell death associated with cytoplasmic vacuolization and is characterized by Rab7- and LAMP1-labeled late endosomes and lysosomes in tumor cells^22^. To define whether *BCV*-triggered cytoplasmic vacuolization cell death in BMDMs is similar to methuosis, we treated BMDMs with MOMIPP, which can cause methuosis in multiple tumor cells (Supplementary Fig. S4a)^22^. Unexpectedly, the cytoplasmic vacuolization induced by MOMIPP in BMDMs but not lung cancer cells A549 was transient and reversible. At 6 h after treatment, MOMIPP-treated BMDMs exhibited remarkable cytoplasmic vacuolization (Supplementary Fig. S4b–d). However, the cytoplasmic vacuolization in BMDMs induced by MOMIPP decreased after 6 h of treatment and almost disappeared after 24 h of continuous treatment (Supplementary Fig. S4b–d). The fluid-phase tracer Lucifer yellow accumulated in vacuoles to form a large punctum in BMDMs during MOMIPP treatment, whereas the distribution of Lucifer yellow in *BCV*-infected BMDMs was restricted to the edge of vacuoles and failed to form big puncta inside the vacuoles (Supplementary Fig. S4e). In line with a previous report, the vacuoles induced by MOMIPP in BMDMs were also labeled with Rab7 and LAMP1 but not EEA1 (Supplementary Fig. S4f). Real-time quantification of cell death and LDH release assay revealed that MOMIPP treatment in BMDMs did not cause dramatic cell death (Supplementary Fig. S4g–i). Collectively, *BCV*-triggered cytoplasmic vacuolization in BMDMs shares common features with methuosis, but is different from the methuosis pathway.

### *BCV*-triggered cytoplasmic vacuolization is a unique cell death pathway

Type I IFN (IFN-I) signaling and inflammasome activation play essential roles in many bacterial pathogen infection-triggered cell death pathways, such as pyroptosis and PANoptosis^4,33,34^. To determine whether IFN-I signaling and ASC-dependent inflammasome activation were involved in the *BCV*-triggered cytoplasmic vacuolization pathway, WT, *Ifnar*^−^^*/*^^−^, *Asc*^−^^*/*^^−^, and *Aim2*^−^^*/*^^−^*Nlrp3*^−^^*/*^^−^ BMDMs were infected with *BC* or *BCV* (Supplementary Fig. S5a). The results showed that the levels of cytoplasmic vacuolization and LDH release induced by *BCV* infection were comparable among these samples (Supplementary Fig. S5a, b). These data indicated that *BCV*-triggered cytoplasmic vacuolization cell death was not dependent on IFN-I signaling or ASC-dependent inflammasome activation.

**Fig. 3:**
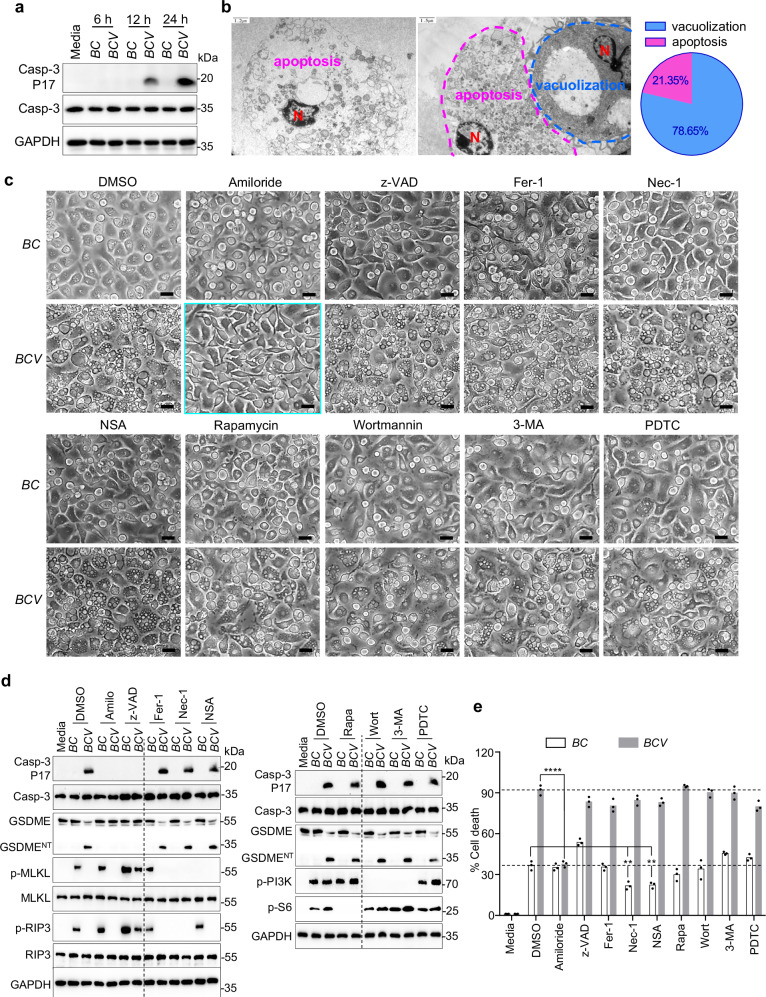
Amiloride inhibits *BCV*-triggered cytoplasmic vacuolization cell death. **a** Immunoblot analysis of caspase-3 and cleaved caspase-3 (P17) in WT BMDMs infected with *BC* or *BCV* (400 MOI) for the indicated time. **b** TEM analysis of WT BMDMs infected with *BCV* (400 MOI) for 30 h (left and middle) and quantification of the percentage of cytoplasmic vacuolization and apoptosis-like cell death within the dead cell population (right). N indicates the cell nucleus. A total of 89 dead cells were analyzed. Scale bars, 1.2 μm for the left and 1.5 μm for the middle. **c** Microscopic analysis of WT BMDMs infected with *BC* or *BCV* (400 MOI) in the presence of amiloride hydrochloride (Amilo, 0.5 μM), z-VAD (25 μM), ferrostatin-1 (Fer-1, 1 mM), necrostatin-1 (Nec-1, 100 μM), necrosulfonamide (NSA, 500 nM), Rapamycin (Rapa, 500 nM), Wortmannin (Wort, 0.2 μM), 3-Methyladenine (3-MA, 5 mM), and pyrrolidinedithiocarbamate ammonium (PDTC, 1 μM) for 12 h. Scale bars, 20 μm. **d** Immunoblot analysis of caspase-3, cleaved caspase-3 (P17), GSDME, cleaved GSDME (GSDME^NT^), p-MLKL, MLKL, p-RIP3, RIP3, p-PI3K, and p-S6 in WT BMDMs infected with *BC* or *BCV* (400 MOI) together with various inhibitors in **c** for 20 h. **e** LDH analysis of WT BMDMs infected with *BC* or *BCV* (400 MOI) in the presence of the indicated inhibitors for 20 h (*n* = 3 biologically independent samples). Data are from 3 independent experiments (**e**) or representative of 3 independent experiments with similar results (**a**–**d**). For **e**, data represent Mean ± SEM, and two-sided Student’s *t*-test without multiple-comparisons correction, **P < 0.01, ****P < 0.0001.

To further examine whether this type of cytoplasmic vacuolization was associated with apoptosis or other types of cell death, we analyzed the hallmarks of different kinds of cell death via protein expression and activation analyses of caspase-3 for apoptosis, caspase-1 for pyroptosis, and phosphorylation of MLKL and RIP3 for necroptosis in *BC*- and *BCV*-infected BMDMs. Indeed, *BCV* infection also triggered caspase-3 activation at later time points but not caspase-1 activation (Fig. 3a; Supplementary Fig. S5c). Notably, TEM analysis of *BCV*-infected BMDMs at later time points revealed that *BCV* infection activated two types of cell death in different cells: major cytoplasmic vacuolization (78.65%) and minor apoptosis-like cell death (21.35%) (Fig. 3b). Cleavage of GSDME by active caspase-3 induces membrane pore formation of the N-terminal domain of GSDME, which mediates the apoptosis switch to pyroptotic cell death^35,36^. Indeed, both caspase-3 activation and downstream GSDME cleavage were observed in *BCV*-infected WT BMDMs (Fig. 3d). *BC* infection induced the phosphorylation of MLKL and RIP3, which might trigger the necroptosis and membrane rupture of *BC*-infected BMDMs (Figs. 2b, 3d).

The apoptosis in bystanders was also reported in necroptosis as a primary cell death event during SARS-CoV-2 infection^37^, suggesting that the apoptosis-like cell death in BMDMs might be a secondary effect of cytoplasmic vacuolization. We found that the supernatant without viable bacteria from *BCV*- but not *BC*-infected BMDMs induced caspase-3 activation (Supplementary Fig. S5d). Thus, we performed mass spectrometry (MS) analysis of supernatants from *BCV*- and *BC*-infected BMDMs and found that lysosomes and lysosome-associated proteins, such as multiple cathepsin proteins, were enriched in the supernatants of *BCV*-treated BMDMs compared with *BC*-treated cells (Supplementary Fig. S5e and Table S3). We purified the lysosomes from *BCV*-infected BMDMs and treated WT BMDMs with purified lysosomes (Supplementary Fig. S5f)^38^. The caspase-3 activation, GSDME cleavage, and apoptosis-like cell death were observed in lysosome-treated BMDMs (Supplementary Fig. S5g, h). Furthermore, cleaved caspase-3 was specifically detected in non-vacuolated BMDMs during *BCV* infection (Supplementary Fig. S5i). Together, these data suggest that lysosomes and lysosome-associated proteinases enriched in the supernatant of *BCV*-infected BMDMs may drive apoptosis-like cell death in bystander cells.

To further examine whether *BCV*-triggered cytoplasmic vacuolization was dependent on other cell death pathways and intracellular events, WT BMDMs were infected with *BC* or *BCV* in the presence or absence of different types of inhibitors, including the pan-caspase inhibitor z-VAD for apoptosis inhibition, the RIPK1 inhibitor necrostatin-1 (Nec-1) and MLKL inhibitor necrosulfonamide (NSA) for necroptosis inhibition, ferrostatin-1 (Fer-1) for ferroptosis inhibition, pyrrolidinedithiocarbamate ammonium (PDTC) for NF-κb inhibition, 3-methyladenine (3-MA) and wortmannin for PI3K and autophagy inhibition, rapamycin for mTORC1 inhibition and autophagy activation, and amiloride hydrochloride for sodium channel inhibition. Notably, amiloride dramatically inhibited *BCV*-triggered cytoplasmic vacuolization, caspase-3 activation, GSDME cleavage, and LDH release (Fig. 3c–e; Supplementary Fig. S5j). Conversely, z-VAD inhibited caspase-3 activation and GSDME cleavage but not cytoplasmic vacuolization or cell death (Fig. 3c–e; Supplementary Fig. S5j), indicating that *BCV*-triggered cytoplasmic vacuolization cell death was not dependent on apoptosis. In addition, inhibitors of necroptosis, ferroptosis, autophagy, mTORC1, or NF-κb did not affect *BCV* infection-triggered cytoplasmic vacuolization, caspase-3 activation, GSDME cleavage, or LDH release (Fig. 3c–e; Supplementary Fig. S5j). The necroptosis inhibitors Nec-1 and NSA inhibited *BC* infection-triggered cell death (Fig. 3d, e). Overall, these results indicated that *BCV* infection induced two separate cell death pathways: cytoplasmic vacuolization and associated apoptosis-like cell death. Apoptosis-like cell death may be a downstream secondary effect of cytoplasmic vacuolization induced by *BCV*, and ion channels are essential for *BCV*-triggered cytoplasmic vacuolization.

### SLC9A9 is important for *BCV*-induced cytoplasmic vacuolization

**Fig. 4:**
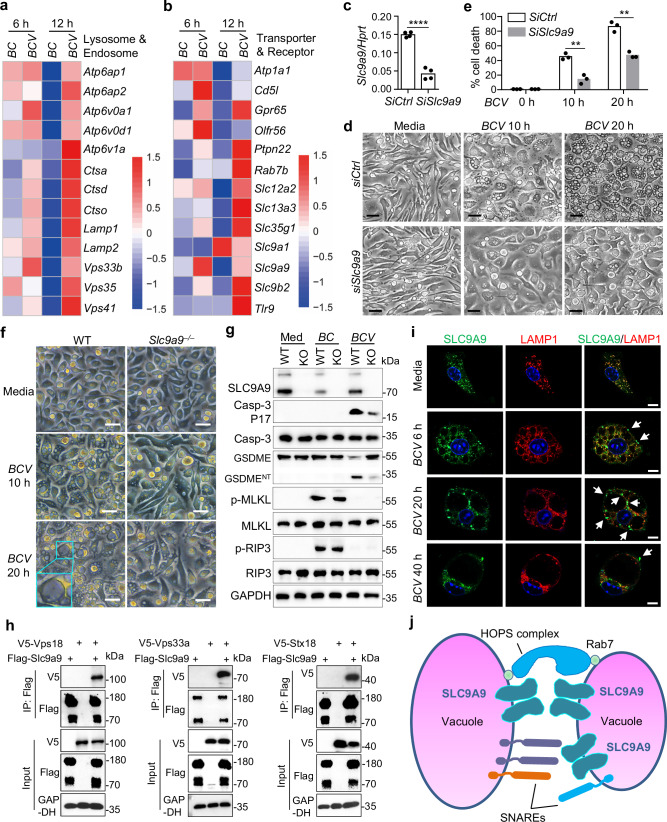
SLC9A9 regulates *BCV* infection-triggered cytoplasmic vacuolization cell death. **a**, **b** RNA-seq analysis of the gene expression of WT BMDMs infected with *BC* or *BCV* (400 MOI) for 6 and 12 h. Heatmap showing the comparison of endosome- and lysosome-associated genes (**a**) and genes encoding receptors and transporters (**b**) between *BC*- and *BCV*-infected BMDMs, with increased expression in *BCV*-infected BMDMs. **c** Quantitative RT-PCR analysis of *Slc9a9* expression in *siCtrl*- and *siSlc9a9*-transfected BMDMs for 36 h (*n* = 4 technical replicates; 3 independent experiments). *Hprt* stands for genes encoding hypoxanthine guanine phosphoribosyltransferase. **d** Microscopic analysis of siRNA-knockdown BMDMs (*siCtrl* and *siSlc9a9*) infected with *BCV* (400 MOI) for the indicated time. Scale bars, 30 μm. **e** LDH analysis of *siCtrl*- and *siSlc9a9*-transfected BMDMs in **d** (*n* = 3 biologically independent samples). **f** Microscopic analysis of WT and *Slc9a9*^−^^*/*^^−^ BMDMs infected with *BCV* (400 MOI) for the indicated time. Scale bars, 30 μm. **g** Immunoblot analysis of caspase-3, cleaved caspase-3 (P17), GSDME, cleaved GSDME (GSDME^NT^), p-MLKL, MLKL, p-RIP3, RIP3, and SLC9A9 in WT and *Slc9a9*^−^^*/*^^−^ (KO) BMDMs infected with *BC* or *BCV* (400 MOI) for 20 h. **h** Immunoblot analysis of Flag-SLC9A9 co-immunoprecipitated with V5-Vps18, V5-Vps33a, and V5-Stx18 from lysates of HEK293T cells transfected with the indicated plasmids. **i** Confocal microscopy analysis of SLC9A9 and LAMP1 in *BCV*-infected (400 MOI) WT BMDMs at the indicated time. The arrows indicate SLC9A9 subcellular localization at the membrane of vacuoles. Scale bars, 10 μm. **j** A hypothetical model of the interaction between SLC9A9 with HOPS, SNAREs and Rab7. Data are from 3 independent experiments (**e**) or representative of 3 independent experiments with similar results (**c**, **d**, **f**–**i**). Data represent Mean ± SEM for **c**, **e**, two-sided Student’s *t*-test without multiple-comparisons correction, **P < 0.01, ****P < 0.0001.

To explore the mechanism underlying *BCV* infection-induced cytoplasmic vacuolization, we performed a genome-wide transcriptional analysis of *BC*- and *BCV*-infected BMDMs. *BCV* infection triggered the enrichment of genes associated with endosomes, lysosomes, and metabolism (Fig. 4a; Supplementary Fig. S6a and Table S4). However, the expression levels of genes involved in inflammatory response pathways, including TNF, chemokine, NF-κB, and MAPK signaling, were dramatically reduced in *BCV*- compared with *BC*-infected BMDMs (Supplementary Fig. S6a–c and Table S4). Given that inhibitors targeting ion channels have suppressive effects on the process of *BCV* infection-induced cytoplasmic vacuolization, we found that specific genes encoding membrane proteins and transporters that were localized to intracellular compartments, especially solute carriers (SLCs), were highly expressed in BMDMs infected with *BCV* but not *BC* (Fig. 4b; Supplementary Fig. S6d).

We performed siRNA transfection for 13 candidate genes more highly expressed in *BCV*-infected than in *BC*-infected BMDMs (Fig. 4b, c; Supplementary Fig. S6e). Notably, the cytoplasmic vacuolization and LDH release induced by *BCV* infection were markedly inhibited in *SiSlc9a9*-transfected BMDMs but not in *SiSlc9a1*-, *SiSlc9b2*-, *SiSlc12a2*-, *SiSlc13a3*-, *SiSlc35g1*-, *SiAtp1a1*-, *SiRab7b*-, *SiGpr65*-, *SiCd5l*-, *SiOlfr56*-, *SiPtpn22*-, or *SiTlr9*-transfected BMDMs (Fig. 4d, e; Supplementary Fig. S6f). To provide genetic evidence for the role of SLC9A9 in the process of *BCV*-triggered cytoplasmic vacuolization, we generated *Slc9a9*^−^^*/*^^−^ mice (Supplementary Fig. S7a–c), which were viable with characteristics similar to those of WT mice. SLC9A9 deficiency in BMDMs significantly reduced the degree of *BCV*-triggered cytoplasmic vacuolization based on microscopy, quantification by 40% Percoll gradient separation, and LDH release assay analyses (Fig. 4f; Supplementary Fig. S7d–f). In addition, the caspase-3 activation and GSDME cleavage induced by *BCV* were substantially reduced in the absence of SLC9A9 (Fig. 4g).

Membrane fusion is mediated by the zipping of soluble *N*-ethylmaleimide sensitive factor attachment protein receptors (SNAREs) and the tethering of homotypic fusion and protein sorting protein complex (HOPS) or class C core vacuole/endosome tethering (CORVET) with the aid of accessory proteins^39–41^. Distinct sets of HOPS-mediated membrane tethering and SNARE-mediated membrane fusion regulate lysosome fusion with late endosomes or autophagosomes and lipid droplet fusion^42,43^. To define whether SLC9A9 promotes vacuole fusion through interacting with membrane fusion machinery, we performed a co-IP analysis to identify SLC9A9-interacting proteins in HEK293T cells transfected with SLC9A9 and other proteins. Notably, SLC9A9 preferentially interacted with all the HOPS subunits Vps11, Vps16, Vps18, Vps33, Vps39, and Vps41 (Fig. 4h; Supplementary Fig. S7g, h). In addition, lysosome-resident Rab7, but not early endosome-associated Rab5a or Rab5b, was demonstrated to interact with SLC9A9 (Supplementary Fig. S7g, h). Furthermore, we detected that SLC9A9 interacted with members within distinct sets of SNARE complexes, including synaptosomal-associated proteins (SNAP) and syntaxins (Stx) proteins. However, Vamp proteins were not detected to interact with SLC9A9 (Fig. 4h; Supplementary Fig. S8a, b). In addition, amiloride treatment inhibited SLC9A9 interaction with Vps11, Vps16, Vps18, Vps33b, Vps39, Rab7, Snap47, and Esyt1 (Supplementary Fig. S9a, b), suggesting that SLC9A9 is one of the targets of amiloride to inhibit cytoplasmic vacuolization. During *BCV* infection in BMDMs, SLC9A9 dramatically localized to the contact sites of LAMP1-associated vacuoles, revealed by confocal microscopy and live-cell imaging analysis (Fig. 4i; Supplementary Fig. S10a, b). However, SLC9A9 deficiency did not affect the formation of cytoplasmic vacuolization triggered by MOMIPP (Supplementary Fig. S10c). Collectively, these data indicate that SLC9A9 acts as an important regulator of vacuole fusion to mediate *BCV*-triggered cytoplasmic vacuolization cell death (Fig. 4j).

### OMVs and barrel-like proteins of *BCV* induce the formation of cytoplasmic vacuolization

**Fig. 5:**
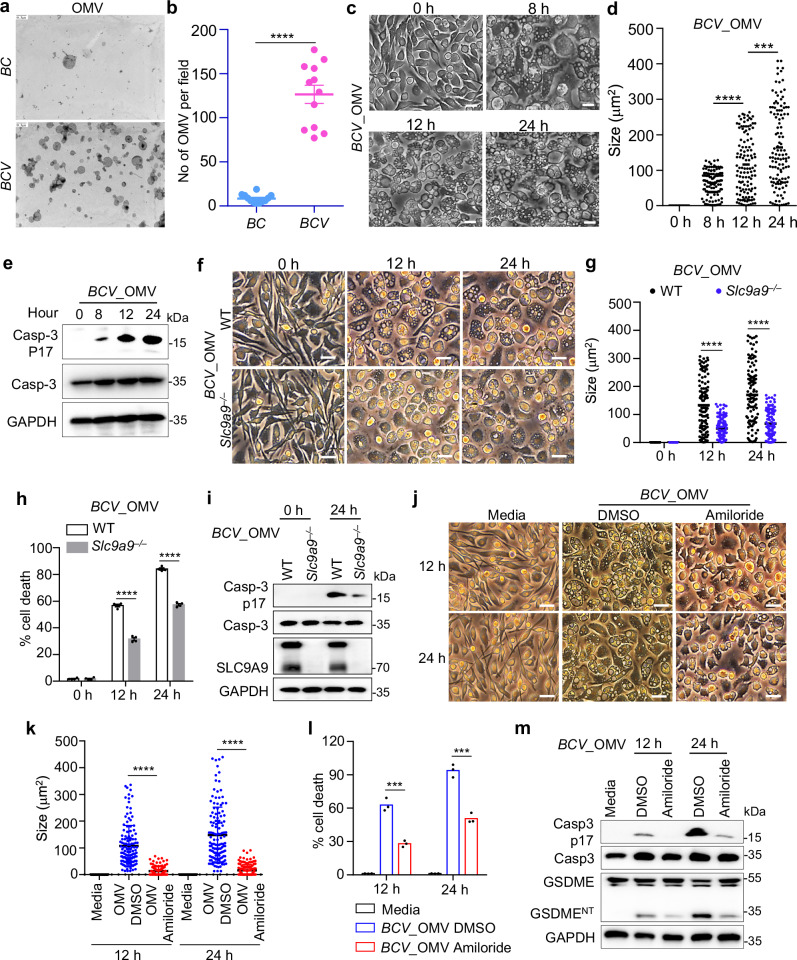
OMVs derived from *BCV* trigger cytoplasmic vacuolization cell death. **a** TEM analysis of purified OMVs from *BC* and *BCV*. *BC* and *BCV* were cultured on Columbia blood agar plates for 96 h, followed by growth in BACTEC™ Lytic media for 16 h. The supernatants of *BC* and *BCV* were used for OMV purification and comparative analysis. Scale bars, 0.3 μm. **b** Quantification of the number of OMVs per field in **a** (*n* = 12 random fields; 3 independent experiments). **c** Microscopic analysis of WT BMDMs treated with OMVs derived from *BCV* (100 μg) for the indicated time. Scale bars, 30 μm. **d** Quantification of vacuole size in the BMDMs in **c**. The largest vacuole per cell was analyzed, and at least 120 cells were quantified for each group. **e** Immunoblot analysis of caspase-3 and cleaved caspase-3 (P17) in WT BMDMs treated with OMVs derived from *BCV* (100 μg) for the indicated time. **f** Microscopic analysis of WT and *Slc9a9*^−^^*/*^^−^ BMDMs treated with OMVs derived from *BCV* (100 μg) for the indicated time. Scale bars, 30 μm. **g** Quantification of vacuole size in the BMDMs in **f**. The largest vacuole per cell was analyzed, and at least 120 cells were quantified for each group. **h** LDH analysis of WT and *Slc9a9*^−^^*/*^^−^ BMDMs treated with OMVs derived from *BCV* (100 μg) for the indicated time (*n* = 4 biologically independent samples). **i** Immunoblot analysis of caspase-3, cleaved caspase-3 (P17), and SLC9A9 in WT and *Slc9a9*^−^^*/*^^−^ BMDMs treated with OMVs derived from *BCV* (100 μg) for the indicated time. **j** Microscopic analysis of WT BMDMs treated with OMVs derived from *BCV* (100 μg) in the presence and absence of amiloride hydrochloride (Amiloride, 0.5 μM) treatment for the indicated time. Scale bars, 30 μm. **k** Quantification of vacuole size in the BMDMs in **j**. The largest vacuole per cell was analyzed, and at least 120 cells were quantified for each group. **l** LDH analysis of WT BMDMs treated with OMVs derived from *BCV* (100 μg) in the presence and absence of amiloride hydrochloride (Amiloride, 0.5 μM) treatment for the indicated time. **m** Immunoblot analysis of caspase-3, cleaved caspase-3 (P17), GSDME, and cleaved GSDME (GSDME^NT^) in WT BMDMs treated with OMVs derived from *BCV* (100 μg) in the presence and absence of amiloride hydrochloride (Amiloride, 0.5 μM) treatment for the indicated time. Data are from 3 independent experiments (**h**, **l**) or representative of 3 independent experiments with similar results (**a**–**g**, **i**–**k**, **m**). Data represent Mean ± SEM for (**b**, **d**, **g**, **h**, **k**, **l**), ***P < 0.001, ****P < 0.0001, by two-sided Student’s *t*-test without multiple-comparisons correction.

Given the difference in membrane puff-ups and blebbing produced by *BC* and *BCV* (Fig. 1e), we examined the OMVs derived from both *BC* and *BCV*. Notably, the production of intact OMVs in *BCV* dramatically increased compared to that in *BC* (Fig. 5a, b). Furthermore, the OMVs derived from *BCV* also triggered cytoplasmic vacuolization and caspase-3 activation (Fig. 5c–e). The cytoplasmic vacuolization cell death triggered by OMVs and downstream caspase-3 activation were substantially reduced in the absence of SLC9A9 (Fig. 5f–i). Furthermore, amiloride treatment also significantly inhibited the cytoplasmic vacuolization, LDH release, caspase-3 activation, and GSDME cleavage of WT BMDMs triggered by OMVs derived from *BCV* (Fig. 5j–m), suggesting that OMVs can mimic *BCV* to induce cytoplasmic vacuolization cell death.

**Fig. 6:**
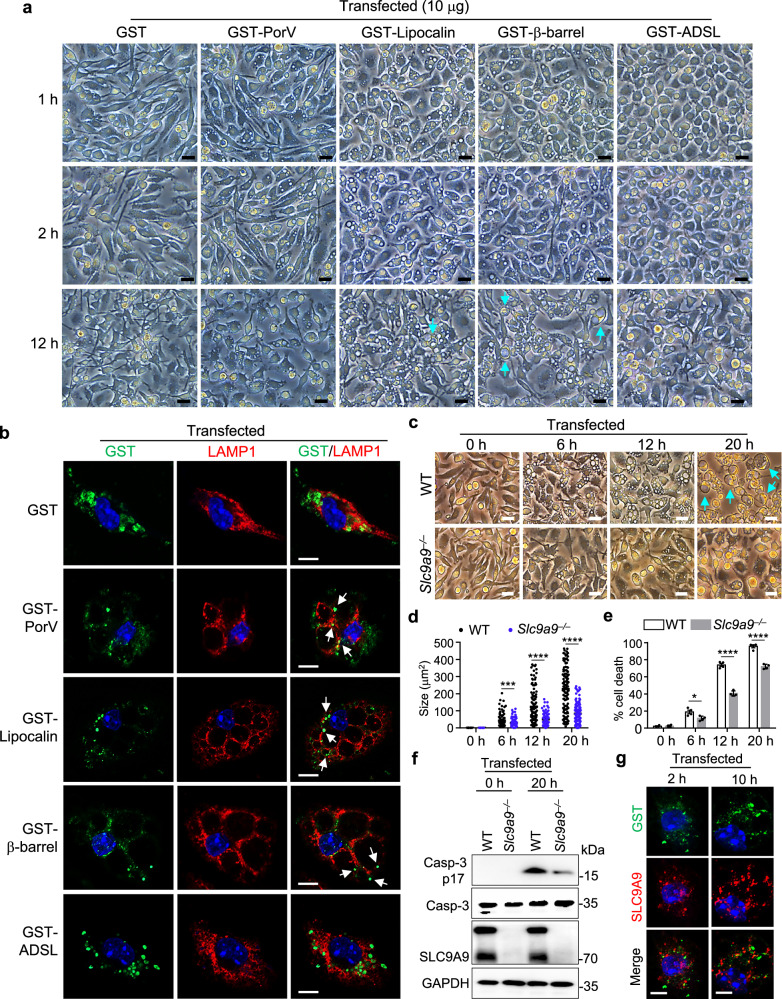
Transfection of the lipocalin, β-barrel, and PorV proteins of *BCV* induces the formation of cytoplasmic vacuolization cell death. **a** Microscopic analysis of WT BMDMs transfected with GST (10 μg), GST-PorV (10 μg), GST-Lipocalin (10 μg), GST-β-barrel (10 μg), and GST-ADSL (10 μg) proteins for 1, 2, and 12 h as indicated. The arrows indicate large vacuole-containing cells. Scale bars, 30 μm. **b** Confocal microscopy analysis of GST and LAMP1 in BMDMs transfected with GST (10 μg), GST-PorV (10 μg), GST-Lipocalin (10 μg), GST-β-barrel (10 μg), and GST-ADSL (10 μg) proteins for 2 h. The arrows indicate the subcellular localization of the GST-fused proteins. Scale bars, 10 μm. **c** Microscopic analysis of WT and *Slc9a9*^−^^*/*^^−^ BMDMs transfected with the combination of GST-Lipocalin (10 μg), GST-β-barrel (10 μg), and GST-PorV (10 μg) proteins for the indicated time. The arrows indicate vacuole-occupied cells. Scale bars, 30 μm. **d** Quantification of vacuole size in WT and *Slc9a9*^−^^*/*^^−^ BMDMs in **c**. The largest vacuole per cell was analyzed, and at least 120 cells were quantified for each group. **e** LDH analysis of WT and *Slc9a9*^−^^*/*^^−^ BMDMs in **c** (*n* = 4 biologically independent samples). **f** Immunoblot analysis of caspase-3, cleaved caspase-3 (P17), and SLC9A9 in WT and *Slc9a9*^−^^*/*^^−^ BMDMs transfected with GST-fused proteins in **c** for 20 h. **g** Confocal microscopy analysis of GST and SLC9A9 in WT BMDMs transfected with the GST-fused proteins in **c** for 2 and 10 h. Scale bars, 10 μm. Data are from 3 independent experiments (**e**) or representative of 3 independent experiments with similar results (**a**–**d**, **f**, **g**). Data represent Mean ± SEM for **d**, **e**, *P < 0.05, ***P < 0.001, ****P < 0.0001, by two-sided Student’s *t*-test without multiple-comparisons correction.

Next, we performed MS analysis of bacterial proteins in the supernatants from *BCV*- and *BC*-infected BMDMs, and detected high levels of PorV, lipocalin, β-barrel protein, and adenylosuccinate lyase (ADSL) in the supernatants of *BCV*-infected BMDMs but not in those of *BC*-infected cells (Supplementary Fig. S11a and Table S3). PorV, lipocalin, β-barrel protein, and adenylosuccinate lyase (ADSL) were also detected in the OMVs derived from *BCV* (Supplementary Fig. S11b and Table S5). However, the expression of *PorV*, *lipocalin*, *β-barrel*, and *ADSL* was comparable between *BCV* and *BC* (Supplementary Table S2), indicating that OMVs derived from *BCV* might contribute to the enrichment of these proteins in the supernatants of *BCV*-infected BMDMs. Thus, we cloned *PorV*, *lipocalin*, *β-barrel*, and *ADSL* from the *BCV* genome into the expression vector and purified the recombinant proteins (Supplementary Fig. S11c). The recombinant proteins of GST-fused PorV, GST-fused lipocalin, GST-fused β-barrel, GST-fused ADSL, or GST alone did not cause cytoplasmic vacuolization following their direct addition to the BMDMs (Supplementary Fig. S11d). However, transfection of GST-fused lipocalin or GST-fused β-barrel proteins into the cells resulted in profound cytoplasmic vacuolization between 1 and 12 h after transfection (Fig. 6a; Supplementary Fig. S12a). The cytoplasmic vacuolization caused by the transfection of the GST-fused PorV protein gradually decreased after 2 h and substantially reduced up to 12 h after treatment (Fig. 6a; Supplementary Fig. S12a). In contrast, transfection of GST-fused ADSL or GST protein alone did not cause cytoplasmic vacuolization in BMDMs (Fig. 6a; Supplementary Fig. S12a).

The intracellular lipocalin, β-barrel, and PorV proteins were preferentially accumulated on the membrane of LAMP1-associated vacuoles (Fig. 6b). Moreover, transfection of the GST-fused lipocalin protein together with the GST-fused β-barrel protein or the combination of the GST-fused lipocalin, GST-fused β-barrel, and GST-fused PorV proteins induced the formation of large vacuoles at 12 h and the formation of single vacuole-occupied cells at 20 h after transfection (Supplementary Fig. S12b, c). In line with *BCV* infection and OMVs treatment, SLC9A9 deficiency significantly inhibited the cytoplasmic vacuolization cell death and downstream caspase-3 activation triggered by the combined transfection of lipocalin, β-barrel, and PorV proteins (Fig. 6c–f). Moreover, amiloride treatment also significantly inhibited the cytoplasmic vacuolization, LDH release, caspase-3 activation, and GSDME cleavage of WT BMDMs transfected with combined lipocalin, β-barrel, and PorV proteins (Supplementary Fig. S12d–g). Protein structure prediction revealed that lipocalin, β-barrel, and PorV had multiple β-strands forming barrel-like structures (Supplementary Fig. S12h). However, the majority of intracellular SLC9A9 distribution did not colocalize with the transfected lipocalin, β-barrel, or PorV proteins (Fig. 6g), indicating that the transfected proteins did not directly interact with SLC9A9 to trigger cytoplasmic vacuolization cell death.

### *BCV* increases in vivo pathogenicity

**Fig. 7:**
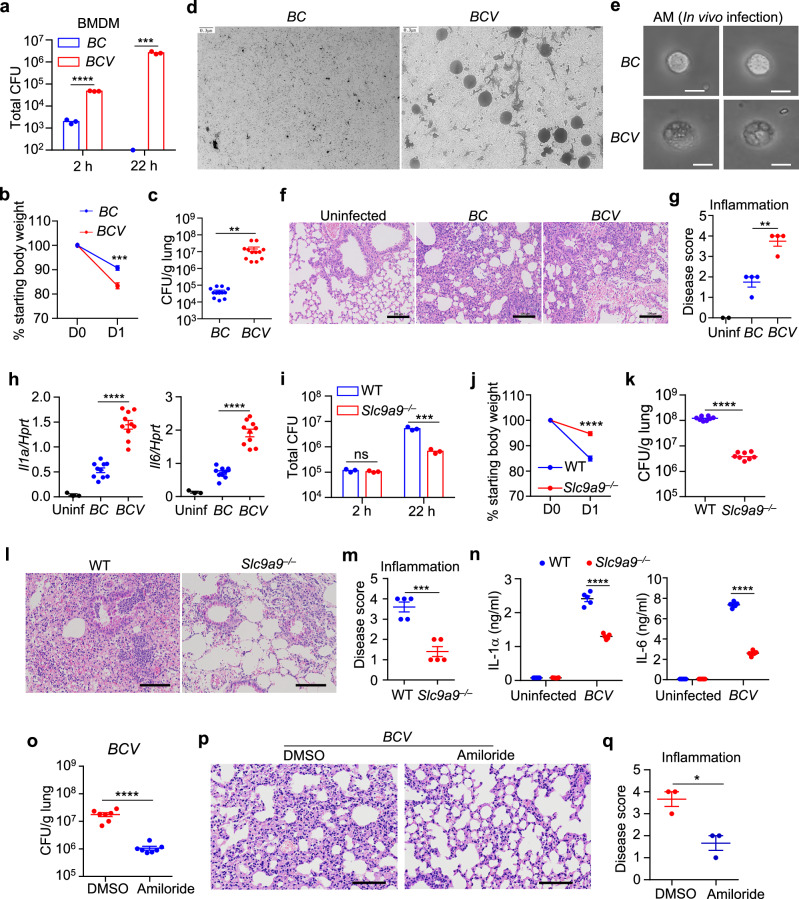
*BCV*-triggered cytoplasmic vacuolization cell death increases pathogenicity in vivo. **a** Bacterial killing ability of *BC* and *BCV* in BMDMs. WT BMDMs were infected with *BC* (20 MOI) and *BCV* (20 MOI) for 2 h, followed by treatment with gentamicin (50 μg/mL) for 1 h. Infected BMDMs were washed, lysed, and cultured on Columbia blood agar plates for 96 h to enumerate intracellular (2 h) bacteria. Washed BMDMs were further cultured in fresh media for 22 h, and the total number of intracellular and extracellular bacteria (22 h) was enumerated after culturing on Columbia blood agar plates for 96 h (*n* = 3 biologically independent samples). **b**, **c** WT female mice were intranasally infected with 4.0 × 10^8^ CFU *BC* (*n* = 12) or *BCV* (*n* = 12), and the body weight change (**b**) and bacterial burden in the lungs on Day 1 after infection were measured (**c**). **d** TEM analysis of OMVs in bronchoalveolar lavage fluid (500 μL volume per mouse) from *BC*- and *BCV*-infected mice in **c**. Scale bars, 0.3 μm. **e** Microscopic analysis of cytoplasmic vacuolization in isolated AMs from *BC*- and *BCV*-infected mice in **c**. Scale bars, 20 μm. **f** H&E staining of lung sections from uninfected and *BC*- and *BCV*-infected mice in **c**. Scale bars, 100 μm. **g** Disease scores based on inflammation in lung sections in **f** from uninfected (Uninf, *n* = 2), *BC*-infected (*n* = 4), and *BCV*-infected (*n* = 4) mice. **h** Expression of genes encoding IL-1α and IL-6 was analyzed in lung tissues from uninfected (Uninf, *n* = 3) and *BC*-infected (*n* = 10), and *BCV*-infected (*n* = 10) mice in **c**. **i** Bacterial killing ability of *BCV* in WT and *Slc9a9*^−^^*/*^^−^ BMDMs. WT and *Slc9a9*^−^^*/*^^−^BMDMs were infected with *BCV* (20 MOI) for 2 h, followed by treatment with gentamicin (50 μg/mL) for 1 h. Infected BMDMs were washed, lysed, and cultured on Columbia blood agar plates for 96 h to enumerate intracellular (2 h) bacteria. Washed BMDMs were further cultured in fresh media for 22 h, and the total number of intracellular and extracellular bacteria (22 h) was enumerated after culturing on Columbia blood agar plates for 96 h (*n* = 3 biologically independent samples). **j**, **k** WT and *Slc9a9*^−^^*/*^^−^ female mice were intranasally infected with 4.0 × 10^8^ CFU *BCV* (*n* = 8 mice for each group), and the body weight change (**j**) and bacterial burden in the lungs on Day 1 after infection were measured (**k**). **l** H&E staining of lung sections from *BCV*-infected mice in **k**. Scale bars, 100 μm. **m** Disease scores based on inflammation in lung sections in **l** from *BCV*-infected WT and *Slc9a9*^−^^*/*^^−^mice (*n* = 5 mice for each group). **n** ELISA analysis of IL-1α and IL-6 in sera from uninfected and *BCV*-infected WT and *Slc9a9*^−^^*/*^^−^mice (*n* = 5 mice for each group) in **k**. **o** WT female mice were intranasally infected with *BCV* (4.0 × 10^8^ CFU), and the bacterial burden in the lungs was measured 24 h after infection. Amiloride indicates that the mice were intravenously injected with amiloride hydrochloride (10 mg/kg) twice, at 0 and 12 h after *BCV* infection (*n* = 7 mice for each group). **p** H&E staining of lung sections from DMSO- or amiloride hydrochloride-treated mice infected with *BCV* in **o**. Scale bars, 100 μm. **q** Disease scores based on inflammation in the lung sections in **p** (*n* = 3 mice for each group). Data are from 2 independent experiments (**j**, **k**) or representative of 3 independent experiments with similar results (**a**–**i**, **l**–**q**). Data represent Mean ± SEM for (**a**–**c**, **g**–**k**, **m**–**o**, **q**), two-sided Student’s *t*-test without multiple-comparisons correction, *P < 0.05, **P < 0.01, ***P < 0.001, ****P < 0.0001.

Confocal microscopy and CFU analysis revealed that the amount of intracellular *BCV* in BMDMs was significantly higher than that of *BC* (Supplementary Fig. S13a–c). The intracellular *BCV* was primarily distributed in the vacuoles labeled with LAMP1 at 8 h post-infection (Supplementary Fig. S13a). After infection in BMDMs, 276 genes were upregulated in *BCV* compared with *BC*, including the genes contributing to stress adaptation, secretion system, invasion, and colonization of bacteria (Supplementary Fig. S13d, e and Table S6). We further performed bacterial killing assay to investigate the bacterial entry and intracellular survival of *BCV* and *BC*, and the results showed that the bacterial entry of *BC* into BMDMs was significantly lower than that of *BCV*, and the bacterial burden of *BCV* at 22 h post-infection was dramatically greater than that of *BC* (Fig. 7a). However, the bacterial growth of *BC* and *BCV* was comparable in culture media (Supplementary Fig. S14a), suggesting that *BCV* evolved the capacity to undergo intracellular propagation.

To examine the pathogenicity of *BCV* and *BC* in vivo, we first infected alveolar macrophages (AMs) isolated from pulmonary lavage fluid ex vivo with *BC* or *BCV*. Consistent with the results obtained using BMDMs, *BCV*-infected AMs demonstrated remarkable cytoplasmic vacuolization (Supplementary Fig. S14b). Next, WT mice were intranasally infected with *BCV* or *BC* (4.0 × 10^8^ CFU per mouse), and the bacterial burden and host response were assessed. Both the body weight loss and bacterial burden in the lungs of the *BCV*-infected mice were significantly greater than those of the *BC*-infected mice 24 h post-infection (Fig. 7b, c). TEM analysis revealed that the OMVs in bronchoalveolar lavage fluid were detected in *BCV*-infected mice but not *BC*-infected mice (Fig. 7d), and cytoplasmic vacuolization in AMs was also observed in *BCV*-infected mice (Fig. 7e). Furthermore, inflammatory responses and lung pathology, including vascular muscle hypertrophy, infiltration of immune cells, and inflammatory cytokine expression, were more severe in the *BCV*-infected than in the *BC*-infected mice (Fig. 7f–h).

To determine the roles of SLC9A9 in *BCV* pathogenesis and host defense, we performed *BCV* infection in WT and *Slc9a9*^−^^*/*^^−^ BMDMs and mice. The intracellular replication of *BCV* in BMDMs was reduced in the absence of SLC9A9 (Fig. 7i). During intranasal infection with *BCV* in WT and *Slc9a9*^−^^*/*^^−^ mice, the body weight loss and bacterial burden in the lungs of the *BCV*-infected *Slc9a9*^−^^*/*^^−^ mice were significantly less than those in WT mice at 24 h post-infection (Fig. 7j, k), Lung pathology and inflammation, and systemic inflammatory cytokines IL-1α and IL-6 in sera were attenuated in the *BCV*-infected *Slc9a9*^−^^*/*^^−^ mice compared with WT mice (Fig. 7l–n). To examine whether immune cells or nonimmune cells contribute to the increased host defense in *Slc9a9*^−^^*/*^^−^ mice during *BCV* infection, we performed bone marrow transplantation between WT (CD45.1^+^) and *Slc9a9*^−^^*/*^^−^ (CD45.2^+^) mice. Flow cytometry analysis revealed that donor-derived bone marrow cells were predominantly detected in the bone marrow, blood, and spleen of recipient mice six weeks after transplantation (Supplementary Fig. S14c), and anti-SLC9A9 western blot analysis showed that SLC9A9 was notably detected in the spleen and lung from recipient *Slc9a9*^−^^*/*^^−^mice that received bone marrow from WT mice, but not detected in the spleen or lung from recipient WT mice that received bone marrow from *Slc9a9*^−^^*/*^^−^ mice (Supplementary Fig. S14d), indicating that the transplantation system is successful in the chimeric mice. Next, chimeric mice were intranasally infected with *BCV*, and the bacterial loads in the lung were determined. Interestingly, WT and *Slc9a9*^−^^*/*^^−^ mice that received bone marrow from *Slc9a9*^−^^*/*^^−^ mice exhibited significantly lower bacterial loads in the lung and less body weight loss than WT and *Slc9a9*^−^^*/*^^−^ mice that received bone marrow from WT mice, respectively (Supplementary Fig. S14e, f), whereas recipient WT and *Slc9a9*^−^^*/*^^−^mice that received same donor cells had a comparable capacity to regulate the bacterial burden and body weight loss (Supplementary Fig. S14e, f). H&E staining revealed that the lung pathology was decreased in WT and *Slc9a9*^−^^*/*^^−^ mice that received bone marrow from *Slc9a9*^−^^*/*^^−^ mice (Supplementary Fig. S14g). These data indicate that SLC9A9 in immune cells plays a dominant role in the host defense against *BCV* infection.

Given that amiloride can inhibit cytoplasmic vacuolization cell death induced by *BCV* in BMDMs, to further determine whether cytoplasmic vacuolization contributed to the high pathogenicity of the *BCV* pathogen, we combined *BCV* administration with amiloride treatment in vivo, which might inhibit the cytoplasmic vacuolization induced by *BCV* infection. Interestingly, the bacterial burden of *BCV* in the lung was significantly reduced in the WT mice treated with amiloride (Fig. 7o). In addition, H&E staining revealed that the lung pathology was decreased in the presence of amiloride (Fig. 7p, q). The amiloride administration in *Slc9a9*^−^^*/*^^−^ mice further increased host defense against *BCV* infection, characterized by less body weight loss, reduced bacterial burden, and decreased lung pathology (Supplementary Fig. S14h–j). Overall, these results indicated that *BCV* was a more virulent variant of the *Bergeyella cardium* bacterial strain, OMVs and the membrane barrel-like proteins lipocalin, β-barrel, and PorV were important for *BCV*-induced cytoplasmic vacuolization cell death via SLC9A9-mediated vacuole fusion (Supplementary Fig. S15).

## Discussion

Previous studies on *Bergeyella cardium* strains are limited to a few clinical case reports due to the difficulty of growth in multiple sets of blood cultures^26,44^. We isolated both *BC* and *BCV* from a patient with infective endocarditis who had used antibiotics long before the clinical examination^26^. Our work demonstrated that *BCV* exhibited several characteristics contributing to immune evasion, including acquired cell entry and intracellular replication capacity, robust OMV release, increased serum killing resistance, downregulated inflammatory cytokine expression, induced cytoplasmic vacuolization cell death, and increased in vivo pathogenicity. Whether the long-term use of antibiotics in patients induced genetic changes or epigenetic switching of *BCV* to promote immune evasion capacity remains unclear. Our study shows *Bergeyella cardium* as an emerging pathogen frequently detected in oral specimens from clinical patients and presents a variant of oral microbiomes has high virulence and is potentially associated with noncausal infectious diseases.

Genomic analysis revealed gene differences between *BC* and *BCV*, including *PorV* and *BatD*. The Bat proteins have been shown to partially compensate for the oxidative stress response in bacteria and provide defense against oxidative damage^45^. In *Francisella tularensis*, the BatD homolog mutant reduced the intracellular replication capacity in macrophages and virulence in a mouse model^46^. Thus, adding the BatD protein to *BCV* might partially contribute to stress adaptation, intracellular replication, and in vivo pathogenicity mechanisms. PorV is a vital machinery component of T9SS and functions as a shuttle protein to deliver T9SS substrates to the attachment complex on the cell surface^47^. Structural prediction revealed that the deletion corresponding to residues 344–351 of *BCV* PorV led to structural changes in PorV. It is possible that the ‘*BCV* β15’ strand promoted β-barrel formation altogether, which might be associated with the robust production of OMVs in *BCV* through mediating the secretion and attachment of cargo proteins to the cell surface and OMVs^48,49^. We speculate that the increased virulence of *BCV* could be partially due to the robust production of OMVs and secretion of barrel-like proteins, causing cytoplasmic vacuolization and cell death. A limitation of this study is the lack of genetic knockout and complementation experiments in *Bergeyella cardium* strains to confirm whether these genomic mutations contributed to robust OMV biogenesis and the secretion of virulence factors in *BCV*. Challenges remain in establishing genetic manipulation techniques for novel organisms due to the lack of genetic manipulation tools^50^, which should be a focus for future development. In this study, *BCV* and *BC* actually represent two mutual mutants of each other for understanding the association between phenotype and genetic differences.

Bacterial OMVs play a critical role in host-microbial interactions that influence pathogenesis by delivering virulence factors and eliciting inflammatory responses^51^. The large number of intact OMVs shed by *BCV* might result in an increasing delivery of virulence factors such as lipocalin, β-barrel, and PorV into BMDMs to cause cytoplasmic vacuolization, whereas the engagement with membrane receptors and internalization were essential for vacuolization triggered by the vacuolating toxins SubAB, CARDS, and VacA^7,10,11^. Lipocalin family members are ancestral proteins found in all kingdoms of life, and secreted lipocalin polypeptides have been reported to induce apoptosis of leukocytes through an autocrine pathway^52^. β-barrel proteins contribute to forming β-barrel outer membrane proteins and are conserved across species in terms of folding and insertion into the outer membrane^53^. Transmembrane β-barrel proteins can fold spontaneously and assemble into lipid membranes to form stable pores^54^. Vacuolating toxin VacA also contains five β-helix subdomains and a C-terminal β-barrel domain^55^. Barrel-like proteins can be delivered into the cytoplasm either through the secretion system of the intracellular *BCV* itself or via *BCV*-derived OMVs. The barrel-like structures of lipocalin, β-barrel, and PorV of *BCV* might contribute to their assembly into the membrane, leading to the initiation and formation of cytoplasmic vacuolization cell death. Defining the receptor’s engagement with the transfected barrel-like proteins to trigger cytoplasmic vacuolization is inspiring.

The interplay between lipids and proteins plays a crucial role in dynamic membrane remodeling, and phosphatidylinositol bisphosphate (PIP_2_) controls the formation and spatiotemporal organization of protein complexes involved in vesicle budding, trafficking, and membrane curvature and fusion^56^. Recently, PIKfyve, a class III phosphoinositide (PI) kinase, was identified as an inhibition target of MOMIPP for inducing methuosis^57^. Interestingly, deficiency of the PIKfyve lipid kinase complex components, including the phosphoinositide (PI) kinase PIKfyve, the scaffolding protein Vac14, and the lipid phosphatase Fig4, inhibited the conversion of phosphatidylinositol-3-phosphate (PI3P) to phosphatidylinositol-3,5-bisphosphate PI(3,5)P_2_ and caused loss of PI(3,5)P_2_ and remarkable lysosome vacuolation in multiple cells in both mice and humans^58–64^. Likewise, PI3P depletion causes the accumulation of deidentified early endosomes, triggering triaptosis^20^. Thus, deidentified early endosome and lysosome might be the direct cause for the formation of vacuolization, and the barrel-like proteins of *BCV* might ultimately influence the homeostasis and distribution of PI(3,5)P_2_ to modulate lysosome membrane dynamics and cytoplasmic vacuolization, which requires further investigation.

The equilibration of osmotic pressure within organelles by water diffusion, rather than programmed gene expression and protein regulation, is involved in transient vacuolization formation induced by natural or synthetic chemical compounds and virulence factors from bacterial and viral pathogens^65–70^. Previously, membrane fusion SNARE proteins Syntaxin 7 and VAMP7 and late endosome docking protein Rab7 were also reported to be essential for clustering late endocytic compartments and cell vacuolization induced by *Helicobacter pylori* VacA treatment^71–73^. This study found that intracellular membrane fusion mediated by SLC9A9 (NHE9) interaction with HOPS components and specific SNARE proteins was critical for the cytoplasmic vacuolization cell death during *BCV* infection. SLC9A9 also colocalizes with intracellular late endosomes and contributes to the transport of sodium and hydrogen ions across the membrane to maintain the pH balance of intracellular organelles^74,75^. Overexpression of SLC9A9 has been reported to lead to endosomal alkalization, SLC9A9 knockdown has been shown to induce endosomal acidification, and the organellar pH is essential for enzyme activity, membrane fusion, and cell volume^74^. In contrast, VacA induced vacuolization mainly through its anion-selective channel for the influx of anions into endosomes and stimulation of the v-ATPase proton pump, which caused the acidification of endosomes^13^. Recently, endosomal PI(3,5)P_2_ was corroborated to directly bind with SLC9A9 for mediating its dimerization and sodium/proton exchange activity^76,77^. Beyond this canonical function, our data revealed that SLC9A9 might principally interact with membrane fusion proteins localized in Rab7-resident vacuoles to initiate assembly of HOPS complex and promote vacuole fusion. However, the interplay between SLC9A9 and the intracellular PI(3,5)P_2_ composition and the balanced regulation between vacuole sodium/proton exchange and the membrane fusion via SLC9A9 remains elusive. SLC9A9 represents one of the significant targets modulated by *BCV* to hijack for immune evasion. Thus, *BCV*-induced vacuolization cell death shares common characteristics with methuosis and VacA-triggered vacuolization of late endosome and lysosome hybrid, but also has distinct features of initiation specificity and involvement of the membrane fusion process. *BCV*-triggered cytoplasmic vacuolization in BMDMs represents a unique cell death, and we term it Fused LysosOme-Associated Termination (Floatptosis).

Solute carriers are a large group of membrane transport proteins with more than 300 members in humans, and the SLC9 family (also called sodium/hydrogen exchanger, NHE) is divided into three subgroups based on membrane localization and cell type specificity^74^. Solute carrier transporters are essential metabolic regulators of immune cells and play critical roles in cancer immunotherapy^78,79^. Dysfunction of SLC9 family members is associated with multiple diseases, such as cancer, neurological disorders, gastrointestinal tract disorders, and kidney disease^80–82^. Thus, the finding of SLC9A9-mediated vacuole fusion and cytoplasmic vacuolization cell death upon *BCV* infection provides novel insights into the mechanisms of both SLC9A9- and vacuolization-associated diseases.

## Experimental procedures

### Mice

*Ifnar*^−^^*/*^^−^, *Aim2*^−^^*/*^^−^*Nlrp3*^−^^*/*^^−^, and *Asc*^−^^*/*^^−^ mice were previously described^83^. *Slc9a9*^−^^*/*^^−^ mice were generated by GemPharmatech Co., Ltd (Suzhou, Jiangsu). Exon 2 of the *Slc9a9* gene was knocked out by CRISPR-Cas9 system. The strategy for constructing the targeting vector is illustrated in Supplementary Fig. S7a. The knockout of the *Slc9a9* gene was validated by genotyping, qRT-PCR, and western blot (Fig. 4g; Supplementary Fig. S7b, c). WT and knockout mice were kept under specific pathogen-free conditions in the Animal Resource Center at Shandong University, Jinan, Shandong Province, China. All animal experiments were conducted in accordance with guidelines approved by the Ethics Committee of Scientific Research of Shandong University.

### Bacteria and OMVs preparation

*BC* and *BCV* were cultured on Columbia blood agar plates (Autobio,0004763) for 96 h, followed by growing in commercial BACTE^TM^ Lytic media from BD BACTEC^TM^ Lytic/10 Anaerobic/F Culture Vials (BD, 442021) with shaking (220 rpm) for 16 h at 37 °C as previously described^26^. The bacteria were precipitated by centrifugation at 3000 rpm for 30 min at 4 °C, and resuspended in DMEM/F-12 media for infection experiments. For OMVs preparation, 200 mL *BCV* culture (OD_600_ is around 1.0) were precipitated by centrifugation at 3000 rpm for 30 min at 4 °C, and the supernatants were passed through a 0.45 µm filter to remove debris, and the filtered supernatants were precipitated by ultracentrifugation at 40,000× *g* for 2 h at 4 °C, and resuspended in 1 mL BACTEC™ Lytic media or PBS for TEM analysis and treatment experiments, respectively. The concentration of OMVs was quantified using Pierce BCA protein assay kit (23227).

### Preparation of BMDMs, treatment, bacterial infection, and siRNA transfection

To generate BMDMs, bone marrow cells were cultured in L929 cell-conditioned DMEM/F-12 supplemented with 10% FBS, 1% nonessential amino acids, and 1% penicillin-streptomycin for 5 days. The siRNAs targeting *Slc9a9*, *Slc9a1*, *Slc9b2*, *Slc12a2*, *Slc13a3*, *Slc35g1*, *Atp1a1*, *Rab7*, *Gpr65*, *Cd5l*, *Olfr56*, *Ptpn22*, and *Tlr9* were ordered from RiboBio Company. siRNAs were electroporated into BMDMs using the Neon^TM^ Transfection System following the manufacturer’s instructions. Briefly, 0.25 nmol siRNAs were electroporated into 5.5 M BMDMs in 110 μL Neon™ Electroporation T Buffer, Electroporation was performed using the Neon™ Transfection System at 2050 V with a single 25 ms pulse. After 36 h, the transfected BMDMs were infected with *BCV* for vacuolization. The siRNA sequences are listed in Supplementary Table S7.

Inhibitors z-VAD (Calbiochem, 627610), necrostatin-1 (Nec-1; Calbiochem, 480065), necrosulfonamide (NSA; MCE, HY-100573), ferrostatin-1 (Fer-1; MCE, HY-100579), pyrrolidinedithiocarbamate ammonium (PDTC; TargetMoI, T3147), 3-Methyladenine (3-MA; APExBIO Technology, A8353), wortmannin (CST, 9951S), rapamycin (MCE, HY-10219), and amiloride hydrochloride (Alomone labs, A-140) were used to treat BMDMs for 2 h with indicated concentration ahead of bacterial infection.

To induce cytoplasmic vacuolization, we infected WT BMDMs in a 12-well plate (1 M per well) with different MOIs of *BCV* (100–400 MOIs) up to 40 h. *BCV*-infected BMDMs were examined by microscopy, and cytoplasmic vacuolization was evaluated according to the number and size of vacuoles using ImageJ software. *BCV* at 400 MOI can induce more cytoplasmic vacuolization and large vacuoles at the early post-infection time period compared with lower MOIs. For comparing experiments, WT, inhibitor-treated, and siRNA-transfected BMDMs were infected with *BC* and *BCV* (400 MOI) as indicated times. The treated and control cells were analyzed for cytoplasmic vacuolization under a microscope and lysed for RNA and protein analysis.

### Bacterial killing assay

BMDMs were infected with *BC* and *BCV* with MOIs of 10–20 for 2 h and treated with antibiotics; cells were washed twice and cultured in fresh media (DMEM/F-12 and 10% FBS). After 22 h, the supernatant (extracellular bacteria) and BMDMs lysed in PBS (intracellular bacteria) were serially diluted, plated onto Columbia Blood agar plates, and incubated for 96 h for CFU enumeration.

### Bacterial infection of mice

*BC* and *BCV* bacterial strains were cultured on Columbia blood agar plates for 96 h, then growing in BACTEC™ Lytic media (BD, 442021) with shaking (220 rpm) for 16 h at 37 °C. Eight- to ten-week-old and gender-matched wild-type mice were infected intranasally with *BC* and *BCV* (4.0 × 10^8^ CFUs per mouse). Mice were weighed and monitored on day 0 and day 1. Mice were euthanized on day 1 after infection, and lungs were harvested to determine the bacterial burden and cytokine expression.

### Immunoblot analysis and antibodies

Samples were separated by 12% SDS-PAGE, followed by electrophoretic transfer to polyvinylidene fluoride membranes, and membranes were blocked and then incubated with primary antibodies. The following primary antibodies were used: anti-caspase-3 (CST, 9662); anti-cleaved caspase-3 (CST, 9661); anti-SLC9A9 (Proteintech, 66577-1-Ig); anti-caspase-1 (AdipoGen, AG-20B-0042); anti-MLKL (Abcepta, AP14272B); anti-p-MLKL (CST, 37333); anti-RIP3 (CST, 95702S); anti-p-RIP3 (CST, 91702S); anti-p-PI3K (CST, 4228); anti-p-S6 (CST, 4856); anti-Cas9 (abcam, ab191468); anti-Flag (Sigma, F3165); anti-V5 (CST, 13202) and anti-GAPDH (CST, 5174). HRP-labeled anti-rabbit (CST, 7074), anti-mouse (CST, 7076), or anti-goat (Santa Cruz, sc-2006) was used as the secondary antibody.

### Immunofluorescence staining and microscopy

For LAMP1, RAB7, SLC9A9, GST, EEA1, RAB5, and cleaved caspase-3 immunostaining, treated and untreated BMDMs were fixed in 4% paraformaldehyde for 15 min at room temperature. Cells were washed with PBS and blocked in 1× ELISA buffer with 0.1% saponin for 1 h. Cells were stained with anti-LAMP1 (Invitrogen, 14-1071-85); anti-RAB7 (CST, 9367); anti-SLC9A9 (Proteintech, 66577-1-Ig), anti-GST (Proteintech, 10000-0-AP), anti-EEA1 (CST,3288), anti-RAB5 (CST, 3574) or anti-cleaved caspase-3 (CST, 9661) — all at 1:300 to 1:500 dilution, overnight at 4 °C. Cells were washed, stained with a fluorescence-conjugated secondary antibody (Invitrogen, A-11008, Alexa Fluor™ 488, Goat anti-Rabbit; Invitrogen, A-21422, Alexa Fluor™ 555, Goat anti-Mouse; Invitrogen, A-11077, Alexa Fluor™568, Goat anti-Rat) at 1:300 dilution for 40 min at 37 °C, and mounted using a mounting medium (Vector Laboratories, H-1200). Lucifer Yellow was ordered from Aladdin (L131282). Cells were observed on the ZEISS-LSM880 and ANDOR High-speed confocal microscope, and image acquisition and data analysis were performed using ZEN black_2-3SP1, ZEN blue 2.6, and Imaris software.

### Live-cell imaging for cell death

BMDMs (0.5 × 10^6^ cells/well) were seeded in 24-well plates. BMDMs were infected with *BC* and *BCV* and stained with propidium iodide (PI; Life Technologies, P3566) and Hoechst (Beyotime Biotechnology, C1029) according to the manufacturer’s instructions. The plate was scanned, and images were acquired in real-time every 2 h from 0 to 24 h post-treatment by Opera Phenix High Content Screening System by PerkinElmer, Inc. Hoechst staining indicates the total number of cells; PI-positive dead cells are marked with a red mask for visualization. The image analysis, masking, and quantification of dead cells were done using the Harmony software package supplied with the system.

### RNA-seq and data analysis

Total RNA was extracted from *BC*- and *BCV*-infected and uninfected WT BMDMs cultured in L929 cell-conditioned DMEM/F-12 media and subjected to commercial RNA-seq analysis (Novogene). Each transcript’s RPKM values (reads per kilobase of transcript per million reads mapped) were calculated. Differential expression analysis between comparative groups was conducted using DESeq2 software (version 1.42.1). Differentially Expressed Genes (DEGs) were selected based on the criteria of |log2(Fold change)| ≥ 1 and a *P*-value less than 0.05. KEGG pathway enrichment analysis of the DEGs was performed using clusterProfiler software (version 4.10.1).

*BC* and *BCV* were cultured on Columbia blood agar plates (Autobio,0004763) for 96 h, and the bacterial RNA was extracted for RNA-seq analysis (LC-BIO Biotech). Differential expression analysis was performed with R package edgeR (version 4.2.0). DEGs were selected based on a logarithmic fold change greater than two and a false discovery rate (FDR) below 0.05. KEGG pathway analyses were conducted using KOBAS to understand the functions of the DEGs. DEGs were considered significantly enriched if their Bonferroni-corrected *P*-value was less than 0.05. The heatmap was plotted using the R package pheatmap version 1.0.12.

### Library preparation, genome sequencing, and assembly

The whole genome of *BC* and *BCV* were sequenced using PacBio Sequel platform and Illumina NovaSeq PE150 at the Beijing Novogene Bioinformatics Technology Co., Ltd. Briefly, the DNA sample was fragmented by sonication to a size of 350 bp, then DNA fragments were end-polished, A-tailed, and ligated with the full-length adapter for Illumina sequencing with further PCR amplification. At last, PCR products were purified (AMPure XP system) and libraries were analyzed for size distribution by Agilent2100 Bioanalyzer and quantified using real-time PCR. The low-quality reads were filtered (less than 500 bp) to improve analysis accuracy and obtain clean data. The long reads were selected by SMRT portal (more than 6000 bp) as the seed sequence, and the other shorter reads were aligned to the seed sequence by Blasr. The SMRT Link software with Illumina data corrected the results of the preliminary assembly. Cyclization was confirmed according to the overlap between the head and the tail, and the initiation site was corrected by the blast with the DNAa database. The National Center for Biotechnology Information (NCBI) did the genome component prediction and gene annotation. The genome sequence dataset has been deposited in GenBank under accession no. CP029149 and CP114055. The genomic alignment between two *BC* and *BCV* was conducted using SnapGene, and scalable circular genome maps were generated using the Java package CGView to display the alignment of the *BCV* genome with the *BC* genome.

### *BC* prevalence analysis in clinical patients

The metagenomic sequencing data of clinical patients used in this study after removing ambient human DNA (150 nt pair-end reads) was obtained from the NCBI public database under accession code PRJNA1057503. The downloaded dataset was subjected to quality control, and low-quality and adapter sequences were removed using TrimGalore (version 0.6.6), and the high-quality sequences were mapped to the *Bergeyella cardium* genome sequence (GenBank CP029149) using BLAT. Sequences that mapped to the reference genome with an alignment rate of ≥ 90% were used for further analysis. Sequences specially mapped to *Bergeyella cardium* genome and not any other species were recognized as potential unique reads of *Bergeyella cardium* by using the kraken2_nt_20230502 and krakenuniq_Standard (20220616) databases^84,85^. Finally, the potential *Bergeyella cardium*-unique sequences were verified using BLASTn against the NCBI nt Database, and the specimens with *Bergeyella cardium*-unique reads above three were classified as *Bergeyella cardium*-positive specimens.

### MS analysis

The supernatants from *BCV*- and *BC*-infected BMDMs, and OMVs derived from *BCV* were performed using mass spectrometry (MS) analysis by Applied Protein Technology (Shanghai). Briefly, protein digestion was performed by trypsin. The digest peptides of each sample were desalted on C18 Cartridges, concentrated by vacuum centrifugation and reconstituted in 40 µL of 0.1% (v/v) formic acid. MS analysis was performed on a timsTOF Pro mass spectrometer (Bruker) coupled to Nanoelute (Bruker Daltonics) for 45 min. The peptides were loaded on a C18-reversed phase analytical column in buffer A (0.1% formic acid) and separated with a linear gradient of buffer B (99.9% acetonitrile and 0.1% formic acid) at a flow rate of 300 nL/min. The mass spectrometer was operated in positive ion mode. The MS raw data for each sample were combined and searched using the MaxQuant software for identification and quantification analysis. Proteins identified in the supernatants from *BCV*- and *BC*-infected BMDMs are listed in Supplementary Table S3. Proteins identified in the OMVs derived from *BCV* are listed in Supplementary Table S5.

### Plasmid construction, transfection and co-IP experiments

The full length of *Slc9a9* was amplified from a mouse cDNA library and subcloned into the pCDH vector. GFP was amplified and subcloned into pCDH-SLC9A9 vector to make GFP-SLC9A9 fusion plasmid. *Lipocalin*, *β-barrel*, *ADSL*, and *PorV* genes were amplified from the DNA of *BCV* and subcloned into the pGEX-6P-2 vector. The full length *Vps11*, *Vps16*, *Vps18*, *Vps33a*, *Vps33b*, *Vps39*, *Vps41*, *Vps35*, *Rab5a*, *Rab5b*, *Rab7*, *Stx2*, *Stx11*, *Stx12*, *Stx18*, *Stxbp1*, *Stxbp2*, *Stxbp3*, *Snap29*, *Snap47*, *Snapin*, *Vamp2*, *Vamp3*, *Vamp4*, *Vamp8*, *Sec22b*, *Bnip1*, *Bet1*, *Bet1l*, *Esyt1*, and *Ehd1* were amplified from a mouse cDNA library and subcloned into pCDNA3.1 vector. All plasmids were confirmed by DNA sequencing. The primer sequences for vector construction are listed in Supplementary Table S7. Lipofectamine 3000 reagents were used for the transient transfection of plasmids into HEK293T cells.

For IP, whole HEK293T cells collected 36 h after transfection were lysed in IP buffer composed of 50 mM Tris-HCl (pH 7.4), 1 mM EDTA, 150 mM NaCl, 1% Triton, and protease/phosphatase inhibitor cocktails (BioTools). After centrifugation, supernatants were collected and incubated with protein A/G Plus–Agrose (Santa Cruz Biotechnology, sc-2003) and 3 μg of the corresponding antibodies for 12 h at 4 °C, followed by 5 rounds of washing with IP buffer. Immunoprecipitated components were eluted in the SDS loading buffer. For immunoblot analysis, immunoprecipitates and input lysates were separated by SDS-PAGE, followed by transfer onto PVDF membranes, and detected by specific antibodies.

### Lentivirus production and infection

The viral particles were prepared by transfecting HEK293T cells with GFP and fusion GFP-SLC9A9 plasmids in combination with packaging vectors. Twelve hours later, the media were replaced with fresh complete DMEM. Viral supernatant was harvested and passed through 0.45 μm syringe filter 48 h and 72 h after transfection. To establish stably transduced cells, WT and *Slc9a9*^−^^*/*^^−^ bone marrows were infected twice with filtered lentiviral supernatant at an MOI of 1.0 in day 3 and day 4 in the presence of polybrene (8 μg/mL) as previously described^86^. The transduced cells were cultured in fresh media for further treatments and analysis.

### Lysosome isolation and treatment

Lysosomes from *BCV*-infected BMDMs (10 M, 18 h) were isolated using a lysosome enrichment kit with cultured cell sonication and density gradient centrifugation, followed by lysosome precipitation according to the manufacturer’s manual (PI89839; Thermo Fisher Scientific). The purity of isolated lysosomes was determined by detecting the lysosomal membrane protein LAMP1, cathepsin B, nuclear protein lamin B, and GAPDH using immunoblotting analysis as previously described^38^. The concentration of lysosome was quantified using Pierce BCA protein assay kit (23227).

### Bone marrow transplantation

The mouse model of bone marrow transplantation was established using WT (CD45.1^+^) and *Slc9a9*^−^^*/*^^−^ (CD45.2^+^) mice as the donors and the recipients, which were similar in age and within 8–12 weeks old as previously described^87^. Six to eight hours prior to transplantation, the recipient mice were irradiated with a dosage of 10 Gy. For transplantation, 10 million bone marrow cells were injected into the tail veins of the recipient mice. Since the second day of transplantation, recipients were fed antibiotics for two weeks. Six weeks after transplantation, bone marrow chimeric mice were used for infection experiments.

### Flow cytometry analysis

For flow cytometric analysis of CD45.1^+^ cells and CD45.2^+^ cells, cells prepared from spleens, bone marrow and peripheral blood were stained using a subset of antibodies (Biolegend, QA18A43, QA18A15, 1:300). Cell preparation and staining with cell surface CD45.1 and CD45.2 were carried out as described previously^87^, and cells were analyzed on a BD LSR Fortessa Cell Analyzer (BD Biosciences).

### Expression, purification, and transfection of recombinant proteins

The control PGEX-6P-2 vector and GST-fused protein constructs PGEX-6P-2-PorV, PGEX-6P-2-Lipocalin, PGEX-6P-2-β-barrel, and PGEX-6P-2-ADSL were transformed into the *Escherichia coli* strain Rosetta (DE3) pLysS for expression. The expressed proteins were loaded onto Glutathione Agarose (MCE) and Mono S^TM^ 5/50 GL (Cytiva, USA) pre-equilibrated with the lysis buffer for purification. Endotoxin was removed using Pierce High-Capacity Endotoxin Removal Resin (Thermo, 88274) according to the manufacturer’s protocol. GST and GST-fused proteins were transfected into the cells using Xfect™ Protein Transfection Reagent (TaKaRa, 631324) following the manufacturer’s instructions.

### Transmission electron microscopy (TEM) and scanning electron microscopy (SEM)

*BC*- and *BCV*-infected BMDMs were fixed in 2% paraformaldehyde and 2.5% glutaraldehyde in 0.1 M cacodylate buffer (pH 7.4) for 1 h at 37 °C. Cells were embedded and sectioned for TEM by Jindi Medical Technology (Jinan, Shandong). *BC* and *BCV* were cultured on Columbia blood agar plates for 96 h, then grown in BACTEC™ Lytic media (BD, 442021) with shaking (220 rpm) for 16 h at 37 °C. An equal number of bacteria were precipitated and fixed in 2.5% glutaraldehyde and phosphate buffer, and TEM and SEM analyzed sectioned samples at Servicebio (Wuhan, Hubei).

### Preparation of tissue sample for HE staining

The superior lobes of the right lungs were fixed in 10% formalin, and 5-μm sections were stained with hematoxylin and eosin (H&E) and examined with a microscope. The severity of lung disease was scored on the basis of the presence of inflammation by a pathologist blinded to the experimental groups according to the grade standard (0 = absent; 1 = rare, minimal; 2 = scattered mild; 3 = multifocal, moderate; 4 = extensive, marked; 5 = severe).

### Real-time qRT-PCR

Total RNA was isolated from cells and tissues using TRIzol reagent (Invitrogen, Thermo Fisher Scientific). cDNA was reverse transcribed using M-MLV reverse transcriptase (Promega). Real-time qRT-PCR was performed on the Roche LightCycler 96 Real-Time Detection System. *Hprt* gene (hypoxanthine guanine phosphoribosyltransferase) is an internal control for data analysis. The primer sequences are listed in Supplementary Table S7.

### Lactate dehydrogenase (LDH) release assay

Cell culture supernatants were collected at the indicated times, and lactate dehydrogenase activity was measured using the Promega cytotoxicity kit (G1781) according to the manufacturer’s protocols.

### ELISA analysis

Sera from uninfected and infected mice were analyzed for cytokine release using ELISA MAX Standard (BioLegend) following the manufacturer’s instructions.

### Protein structure prediction and analysis

The protein structures of Lipocalin, β-barrel, PorV, and ADSL were predicted by the online server trRosetta (https://yanglab.qd.sdu.edu.cn/trRosetta/)^29,88,89^, and visualized by UCSF ChimeraX^90^. Briefly, the protein sequence was used as the input sequence for structure prediction, performed with default parameter settings. trRosetta provided five models as results for each prediction. The highest-ranked model of each prediction, model 1, was used for further analysis. The confidence of the overall structure prediction is reflected by the TM-score. A TM-score (0–1) above 0.5 usually indicates a model with correct topology. The confidence of the prediction at each residue is indicated by per-residue LDDT scores, ranging from 0 to 100, located at the B-factor column of the PDB file of the structure. For structure analysis and comparison of *BC* and *BCV* PorV, structural figures were made using Open-Source PyMOL (Schrödinger). *BCV* and *BC* PorV models are colored by the ‘b-factor’ ‘spectrum’, reflecting the per-residue LDDT scores. The structure model of *BC* PorV was superposed onto *BCV* PorV using the ‘super’ command in PyMOL, resulting in an RMSD of 0.489 over 1961 atoms.

### Statistics

Data are presented as the mean ± SEM. Statistical analyses were performed using two-way ANOVA, two-tailed Student’s *t*- and log-rank tests. *P*-values of 0.05 or less were considered significant.

### Study approval

The study was approved by the Ethics Committee of Scientific Research of Shandong University (ECSBMSSDU2021-2-171 and ECSBMSSDU2021-1-086), Jinan, Shandong Province, China.

## Supplementary information

Supplementary figures

Supplementary Video S1

Supplementary Table S1

Supplementary Table S2

Supplementary Table S3

Supplementary Table S4

Supplementary Table S5

Supplementary Table S6

Supplementary Table S7
