# Supplementary material for: Bergeyella cardium variant induces a unique cytoplasmic vacuolization cell death floatptosis in macrophage
Source: Cell Discov. 2025 Oct 21;11:83. doi: 10.1038/s41421-025-00840-x (PMC12537989; doi:10.1038/s41421-025-00840-x)
Supplement: Supplementary file 1 — Supplementary figures [file 41421_2025_840_MOESM1_ESM.pdf]

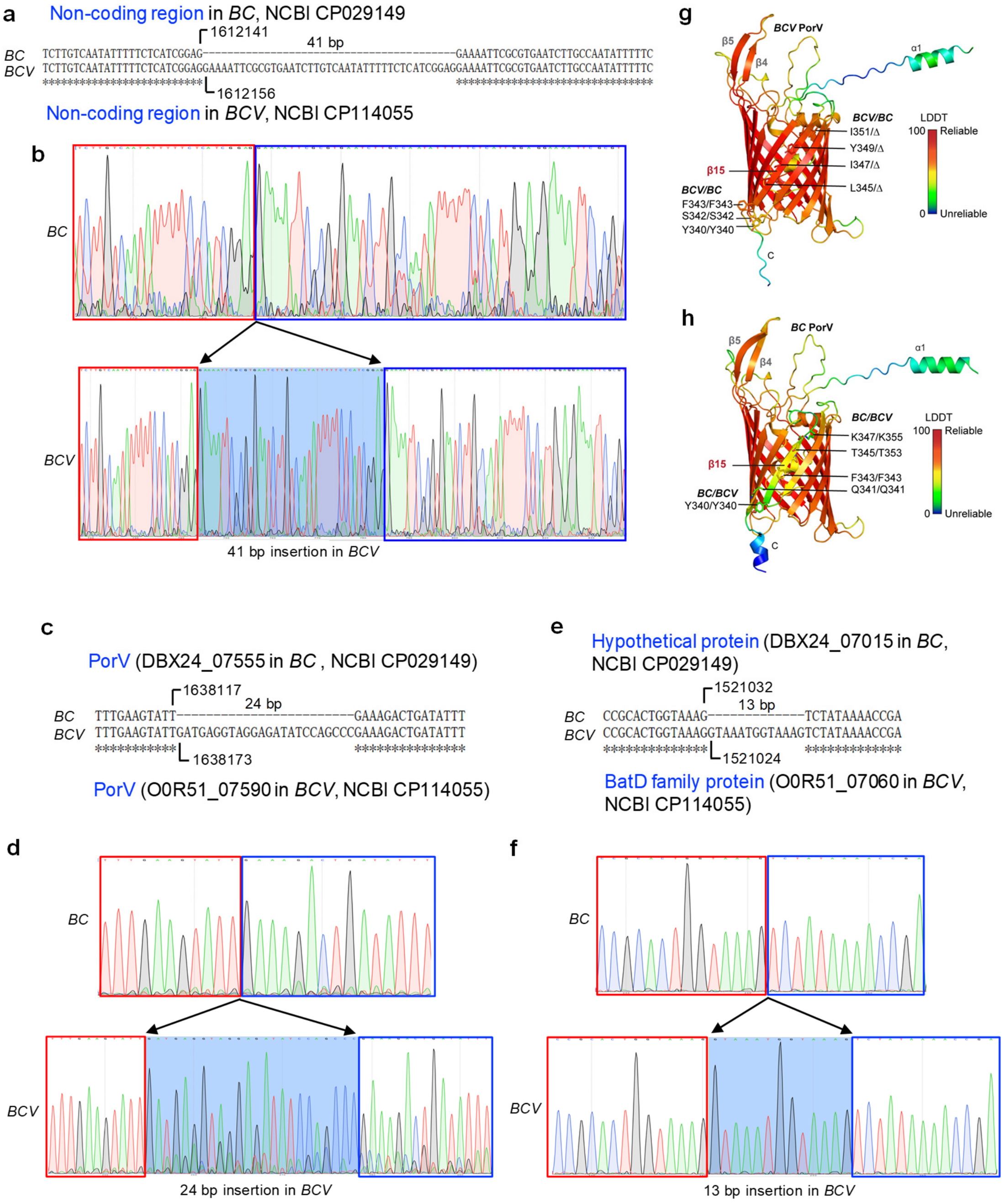

Supplementary Figure S1. Validation of the genomic differences between *BC* and *BCV*.

a Sequence Alignment by CLUSTALW of the non-coding region from the genomes of *BC* (CP029149) and *BCV* (CP114055). The number indicates the sequence position within the genome.

b Validation of the difference in **a** by Sanger sequencing.

c Sequence Alignment by CLUSTALW of *PorV* gene from the genomes of *BC* (CP029149) and *BCV* (CP114055). The number indicates the sequence position within the genome.

d Validation of the difference in **c** by Sanger sequencing.

e Sequence Alignment by CLUSTALW of the hypothetical gene from the genomes of *BC* (CP029149) and *BCV* (CP114055). The number indicates the sequence position within the genome.

f Validation of the difference in **e** by Sanger sequencing.

g Structural model of the *BCV* PorV predicted by trRosseta. The structure is colored with a spectrum from blue to red, according to LDDT values (0-100), which reflects the reliability of the prediction at each residue. The overall estimated TM-score is 0.915. The '*BCV* β15' is labeled as β15, and the sidechains for '*BCV* β15' and nearby residues are shown as sticks. The residue numbers of *BCV* and *BC* PorV are both indicated for comparison. Δ means the residue is deleted.

h Structural model of the *BC* PorV predicted by trRosseta. The model was superposed onto *BCV* PorV and shown in the same orientation as *BCV* PorV. The color scheme is the same as in **g**. The overall estimated TM-score for *BC* PorV is 0.899.

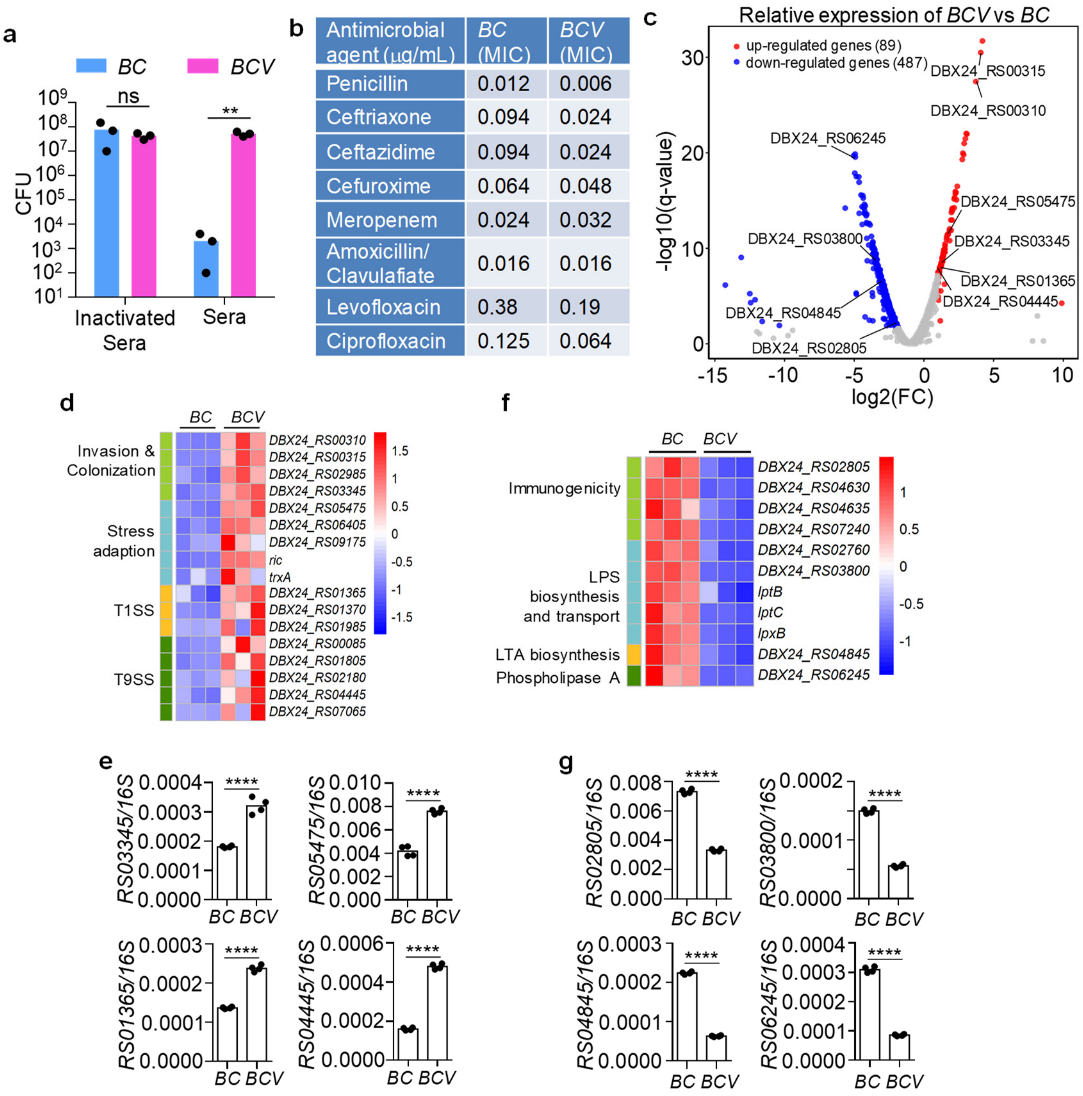

b

Antimicrobial agent (μg/mL)	BC (MIC)	BCV (MIC)
Penicillin	0.012	0.006
Ceftriaxone	0.094	0.024
Ceftazidime	0.094	0.024
Cefuroxime	0.064	0.048
Meropenem	0.024	0.032
Amoxicillin/ Clavulafiate	0.016	0.016
Levofloxacin	0.38	0.19
Ciprofloxacin	0.125	0.064

Supplementary Figure S2. Serum killing and gene expression analysis of *BC* and *BCV*.

a Colony formation analysis of *BC* and *BCV* treated with human sera. Sera were inactivated at 56 °C for 30 min. *BC* and *BCV* were cultured on Columbia blood agar plates for 96 h, followed by growth in BACTEC™ Lytic media for 16 h, and $1x10^8$ CFU of *BC* and *BCV* were incubated with normal and heat-inactivated sera for 2 h at 37 °C. Sera-treated *BC* and *BCV* were grown on Columbia blood agar plates for 96 h, and the numbers of bacteria were enumerated. Each dot represents an individual experiment (n = 3 biologically independent samples).

b Minimum inhibitory concentration (MIC) analysis of *BC* and *BCV* in the presence of different antimicrobial agents.

c RNA-seq analysis of gene expression in *BC* and *BCV*. *BC* and *BCV* were cultured on Columbia blood agar plates for 96 h, after which RNA-seq analysis was performed. Volcano plot showing the distribution of upregulated (red) and downregulated (blue) genes at the transcriptional level in *BCV* compared with *BC*.

d Heatmap analysis of genes highly expressed in *BCV* but not in *BC*.
e Quantitative RT-PCR analysis of *RS03345*, *RS05475*, *RS01365*, and *RS04445* expression in *BC* and *BCV* strains (*n* = 4 technical replicates; 3 independent experiments). *16S* rRNA was used as an internal control to normalize the bacterial gene expression.
f Heatmap analysis of genes highly expressed in *BC* but not in *BCV*.
g Quantitative RT-PCR analysis of *RS02805*, *RS03800*, *RS04845*, and *RS06245* expression in *BC* and *BCV* strains (*n* = 4 technical replicates; 3 independent experiments). *16S* rRNA was used as an internal control to normalize the bacterial gene expression.
Data are from 3 independent experiments (**a**, **c**, **d**, **f**) or representative of 3 independent experiments with similar results (**b**, **e**, **g**). Data represent Mean ± SEM for (**a**, **e**, **g**), **P < 0.01, ****P < 0.0001, by two-sided Student's *t*-test without multiple-comparisons correction.

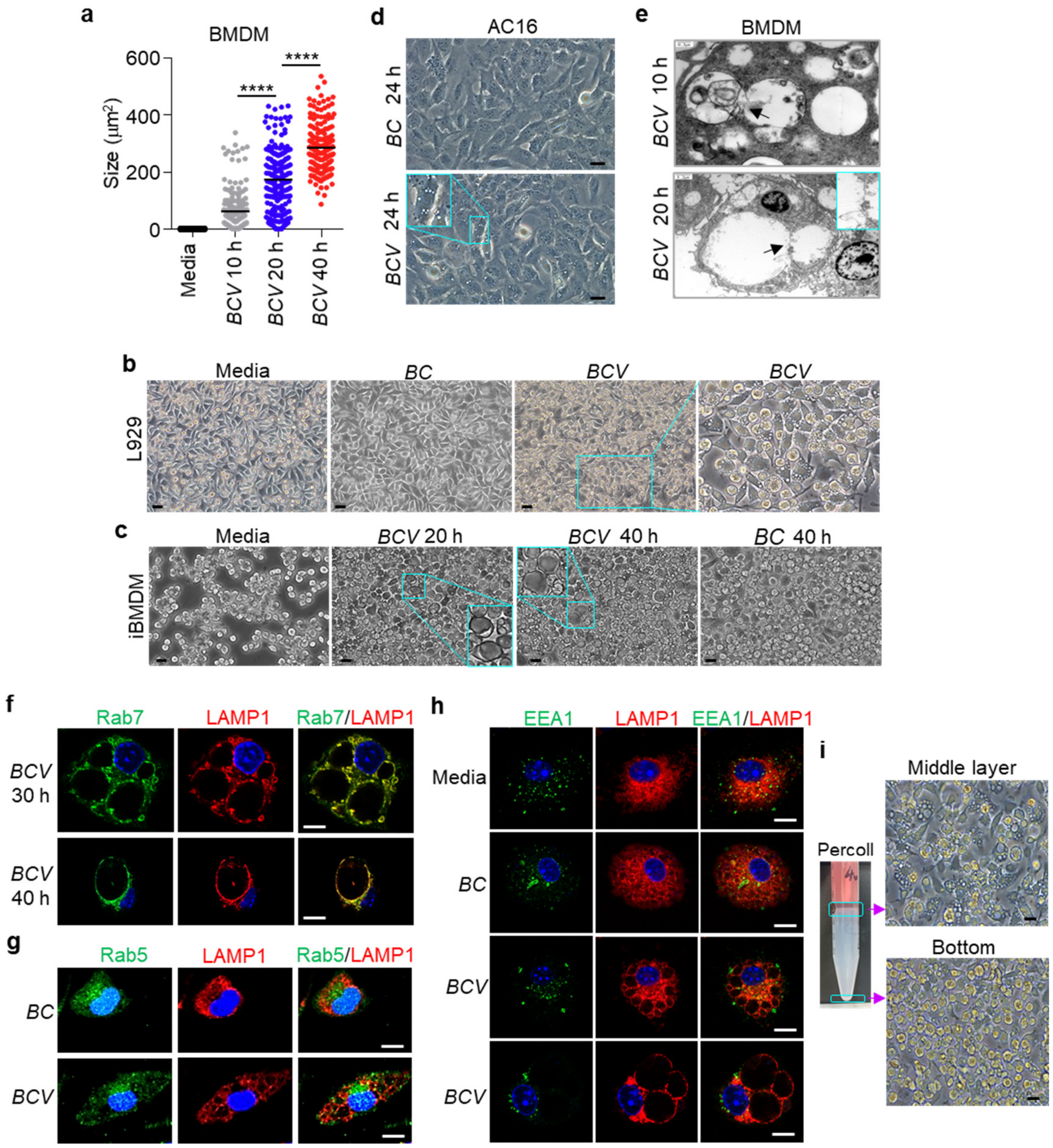

Supplementary Figure S3. Characterization of *BCV* infection-induced cytoplasmic vacuolization cell death.

a Quantification of vacuole size in WT BMDMs infected with *BCV* (400 MOI) for the indicated time in (Fig. 2a). The largest vacuole per cell was analyzed, and 200 cells were quantified for each group.

b Microscopic analysis of L929 cells infected with *BC* or *BCV* (400 MOI) for 12 h. Scale bars, 20 μm.

c Microscopic analysis of iBMDMs infected with *BC* or *BCV* (400 MOI) for the indicated time. Scale bars, 20 μm.

d Microscopic analysis of AC16 infected with *BC* or *BCV* (400 MOI) for 24 h. Scale bars, 20 μm.

e TEM analysis of WT BMDMs infected with *BCV* (400 MOI) for the indicated time. The arrows indicate the membrane fusion and an enlarged image is

shown.
f Confocal microscopy analysis of Rab7 and LAMP1 in *BCV*-infected (400 MOI) WT BMDMs for the indicated time. Scale bars, 10 μm.
g Confocal microscopy analysis of Rab5 and LAMP1 in *BC*- or *BCV*-infected (400 MOI) WT BMDMs for 20 h. Scale bars, 10 μm.
h Confocal microscopy analysis of EEA1 and LAMP1 in uninfected (Media) and *BC*- or *BCV*-infected (400 MOI) WT BMDMs for 20 h. Scale bars, 10 μm.
i Microscopic analysis of the mixture of uninfected and *BCV*-infected BMDMs (400 MOI, 20 h) separated by 40% Percoll gradient. Scale bars, 20 μm.
Data are representative of 3 independent experiments with similar results. Data represent Mean ± SEM for (**a**), ****P < 0.0001, by two-sided Student's *t*-test without multiple-comparisons correction.

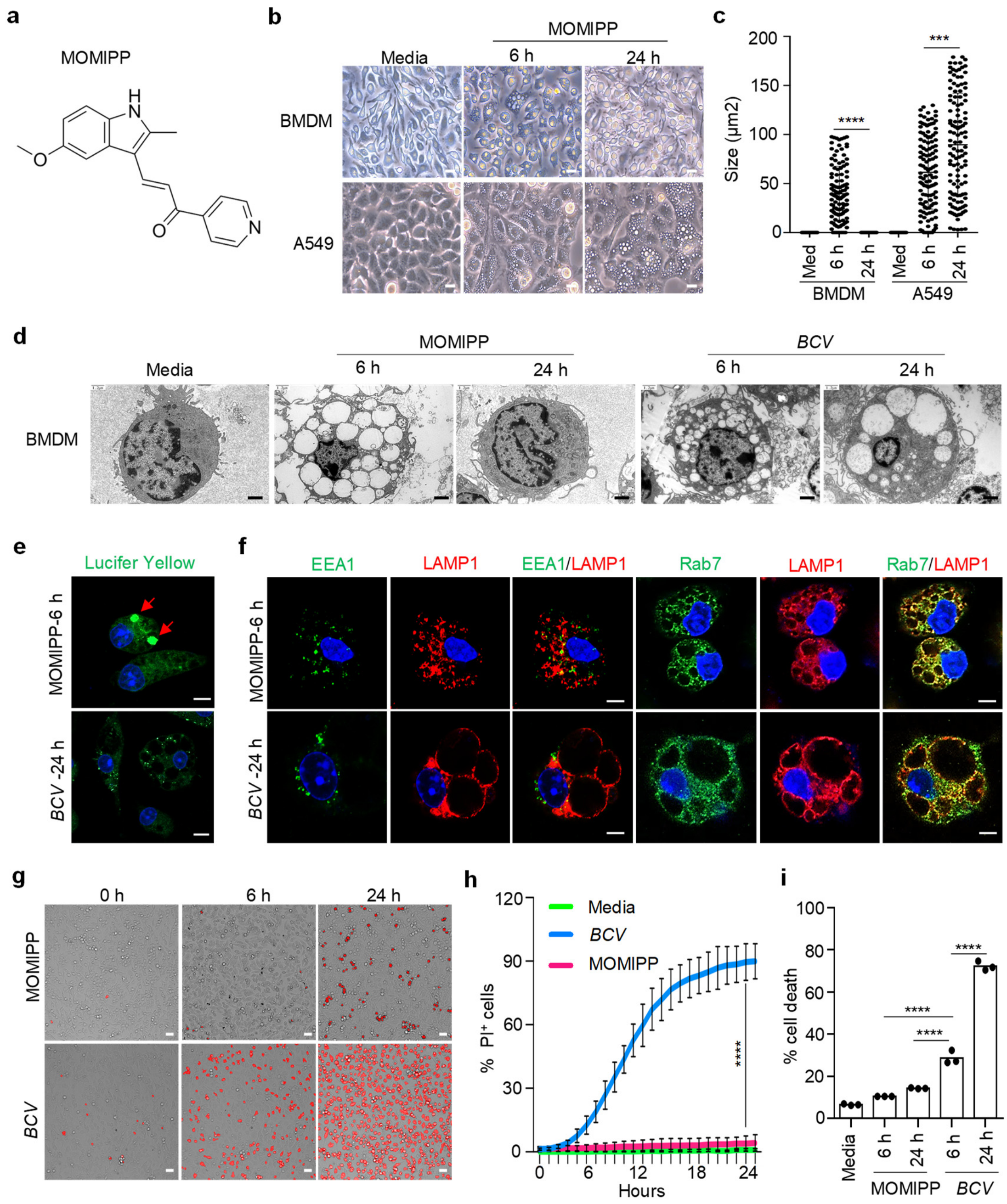

Supplementary Figure S4. MOMIPP-induced methuosis in BMDMs is a transient cytoplasmic vacuolization.

a Molecular structure of MOMIPP.

b Microscopic analysis of WT BMDMs and A549 cells treated with MOMIPP (3 μM) for the indicated time. Scale bars, 30 μm.

c Quantification analysis of vacuole size in WT BMDMs and A549 cells treated with MOMIPP (3 μM) in **b**. The largest vacuole per cell was analyzed, and at least 130 cells were quantified for each group.

d TEM analysis of WT BMDMs treated with MOMIPP (3 μM) or infected with *BCV* (400 MOI) for the indicated time. Scale bars, 1.2 μm.

e Confocal microscopy analysis of Lucifer Yellow (0.5 mg/mL) in MOMIPP-

treated (3 μM) and *BCV*-infected (400 MOI) WT BMDMs for the indicated time. Arrows indicate the vacuoles occupied with Lucifer Yellow. Scale bars, 10 μm.
f Confocal microscopy analysis of EEA1, Rab7, and LAMP1 in MOMIPP-treated (3 μM) and *BCV*-infected (400 MOI) WT BMDMs for the indicated time. Scale bars, 10 μm.
g Representative images of PI staining in untreated, MOMIPP-treated (3 μM), and *BCV*-infected (400 MOI) BMDMs for the indicated time. Scale bars, 20 μm.
h Real-time quantitative live-cell imaging and analysis of cell death in uninfected WT BMDMs and WT BMDMs treated with MOMIPP (3 μM) or infected with *BCV* (400 MOI) (n=10 random fields; 3 independent experiments).
i LDH analysis of WT BMDMs treated with MOMIPP (3 μM) or infected with *BCV* (400 MOI) for the indicated time (*n* = 3 biologically independent samples). Data are from 3 independent experiments (**i**) or representative of 3 independent experiments with similar results (**b**-**h**). Data represent Mean ± SEM for (**c**, **i**), by two-sided Student's *t*-test without multiple-comparisons correction, and two-way ANOVA for (**h**). ***P < 0.001, ****P < 0.0001.

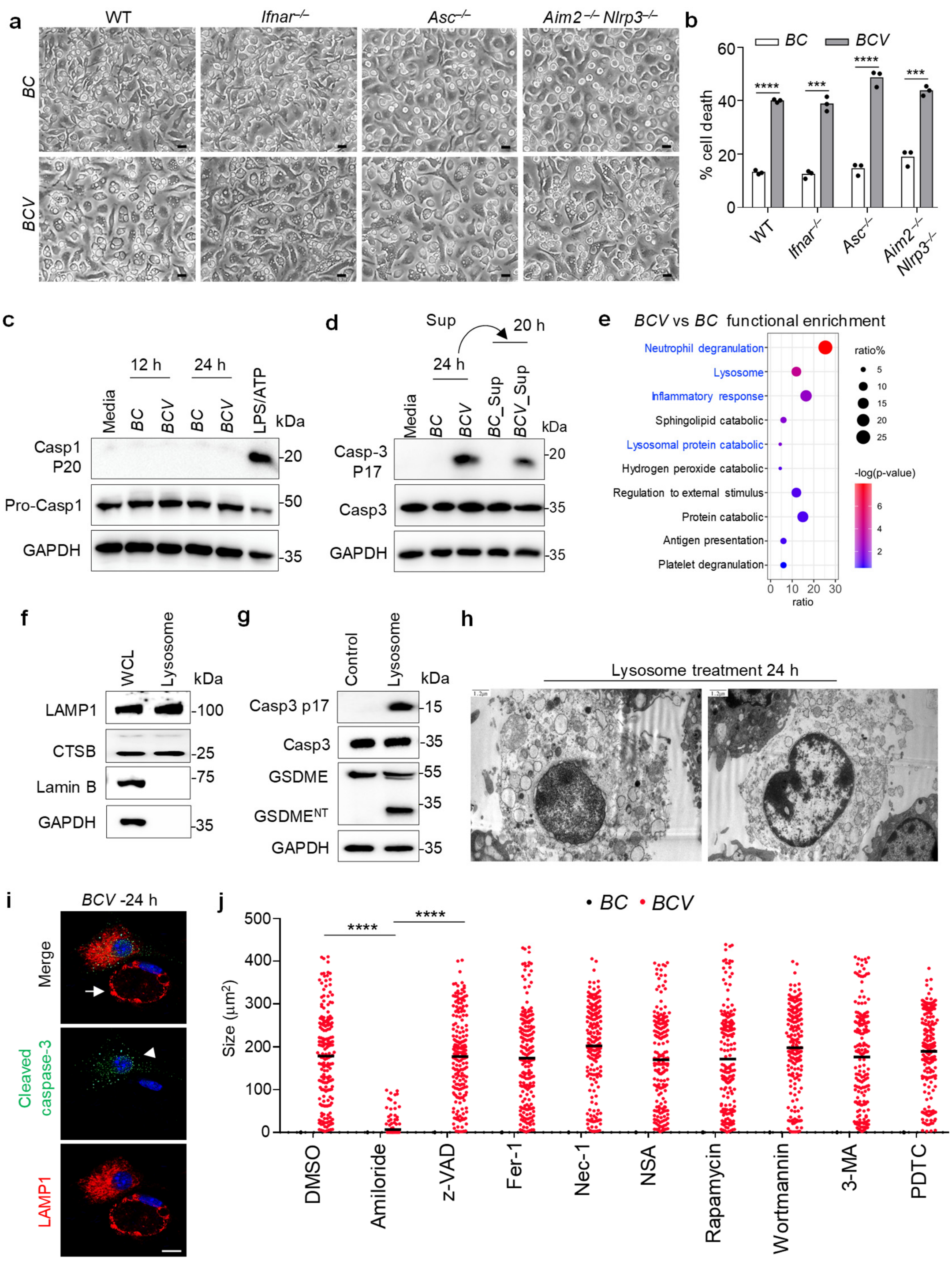

Supplementary Figure S5. Characterization of cell death induced by lysosomes purified from *BCV*-infected BMDMs.

a Microscopic analysis of WT, *Ifnar*$^{-/-}$, *Asc*$^{-/-}$, *and Aim2*$^{-/-}$*Nlrp3*$^{-/-}$ BMDMs infected with *BC* or *BCV* (400 MOI) for 12 h. Scale bars, 20 μm.

b LDH analysis of WT, *Ifnar*$^{-/-}$, *Asc*$^{-/-}$, *and Aim2*$^{-/-}$*Nlrp3*$^{-/-}$ BMDMs infected with *BC* or *BCV* (400 MOI) for 12 h (*n* = 3 biologically independent samples).

c Immunoblot analysis of caspase-1 and cleaved caspase-1 (P20) in WT BMDMs infected with *BC* or *BCV* (400 MOI) for the indicated time. LPS and ATP treatment is a positive control for activating the NLRP3 inflammasome.
d Immunoblot analysis of caspase-3 and cleaved caspase-3 (P17) in WT BMDMs infected with *BC* or *BCV* (400 MOI) for 24 h or treated with the supernatants (Sup) from *BC*- and *BCV*-infected BMDMs for 20 h. Supernatants were passed through 0.22 μm filer and centrifugated to remove viable bacteria and debris.
e Enrichment analysis of host proteins in supernatants from *BCV*- or *BC*-infected (400 MOI) BMDMs for 20 h detected by MS.
f Immunoblot analysis of LAMP1, cathepsin B (CTSB), and Lamin B in whole cell lysate (WCL) and lysosomes purified from *BCV*-infected BMDMs (400 MOI, 20 h).
g Immunoblot analysis of caspase-3, cleaved caspase-3 (P17), GSDME, and cleaved GSDME ($GSDME^{NT}$) in WT BMDMs treated with lysosomes purified from *BCV*-infected BMDMs (100 μg) for 24 h.
h TEM analysis of WT BMDMs treated with purified lysosomes (100 μg) for 24 h in **g**. Scale bars, 1.2 μm.
i Confocal microscopy analysis of cleaved caspase-3 and LAMP1 in WT BMDMs infected with *BCV* (400 MOI) for 24 h. The arrow indicates a vacuolated cell, and the arrowhead indicates a cleaved caspase-3-positive cell. Scale bar, 10 μm.
j Quantification of vacuole size in BMDMs in (Fig. 3c). The largest vacuole per cell was analyzed, and at least 200 cells were quantified for each group.
Data are representative of 3 independent experiments with similar results (**a**-**j**). Data represent Mean ± SEM for (**b**, **j**), ***$P < 0.001$, ****$P < 0.0001$, by two-sided Student's *t*-test without multiple-comparisons correction.

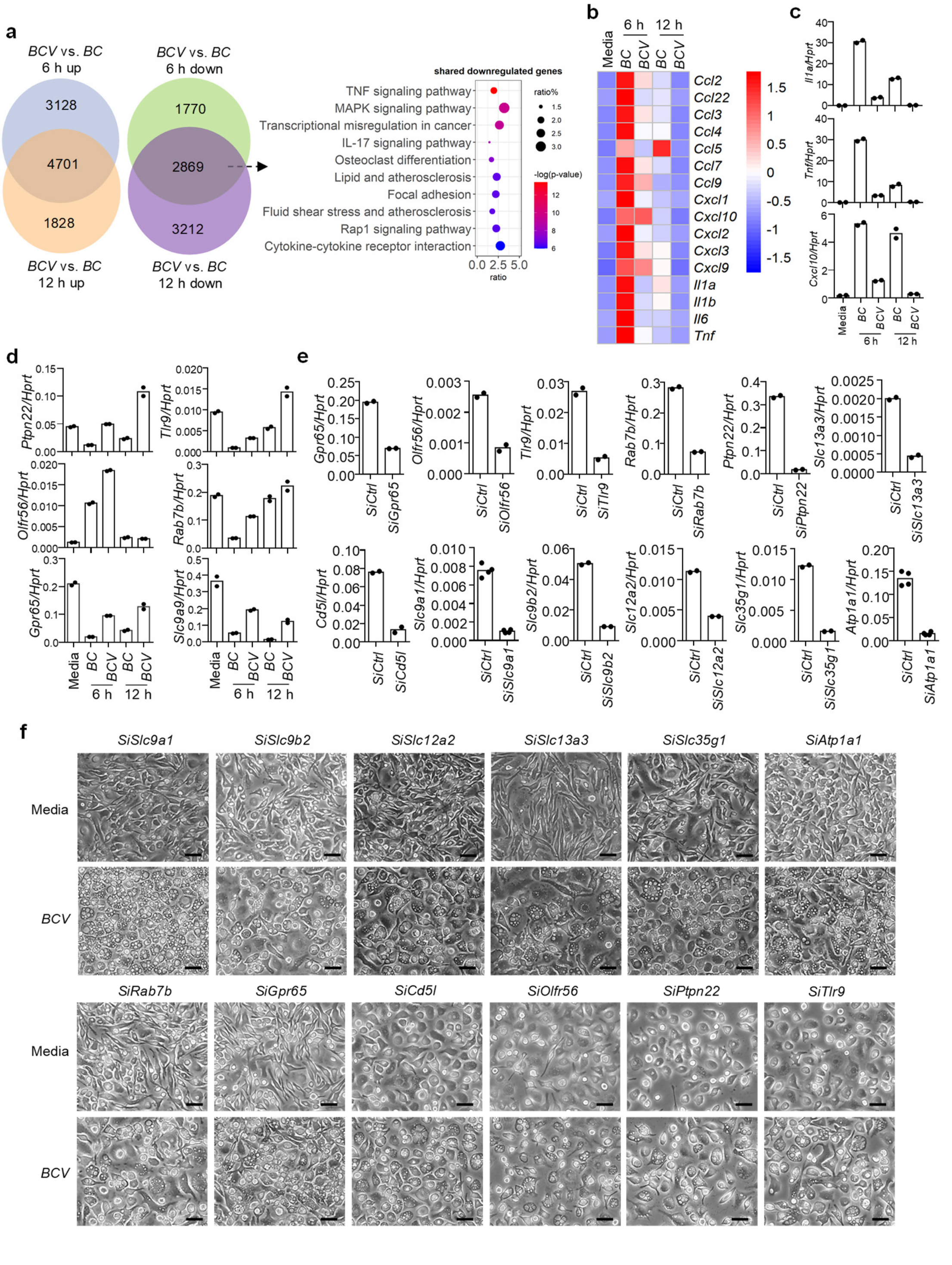

Supplementary Figure S6. The inflammatory response is downregulated in *BCV*-infected BMDMs compared with *BC*-infected BMDMs.

a Enrichment analysis of the genes highly expressed in *BCV*- and *BC*-infected BMDMs. The top 10 enriched pathways of downregulated DEGs (*BCV* vs *BC*) are displayed.

b Heatmap analysis of genes encoding cytokines and chemokines with decreased expression in *BCV*-infected BMDMs.

c Quantitative RT-PCR analysis of *Il1a*, *Tnf*, and *Cxcl10* expression in uninfected (Media) and *BC*- and *BCV*-infected BMDMs for the indicated time (*n* = 2 technical replicates; 3 independent experiments).
d Quantitative RT-PCR analysis of *Ptpn22*, *Tlr9*, *Olfr56*, *Rab7b*, *Gpr65*, and *Slc9a9* expression in uninfected (Media), and *BC*- or *BCV*-infected BMDMs for the indicated time (*n* = 2 technical replicates; 3 independent experiments).
e Quantitative RT-PCR analysis of *Gpr65*, *Olfr56*, *Tlr9*, *Rab7b*, *Ptpn22*, *Cd5l*, *Slc9a1*, *Slc9b2*, *Slc12a2*, *Slc13a3*, *Slc35g1*, and *Atp1a1* expression in *siRNAs* transfected BMDMs for 36 h as indicated (*n* = 2 or 4 technical replicates; 3 independent experiments).
f Microscopic analysis of siRNAs-knockdown BMDMs infected with *BCV* (400 MOI) for 20 h. Scale bars, 30 μm.
Data are representative of 3 independent experiments with similar results (**c**-**f**).

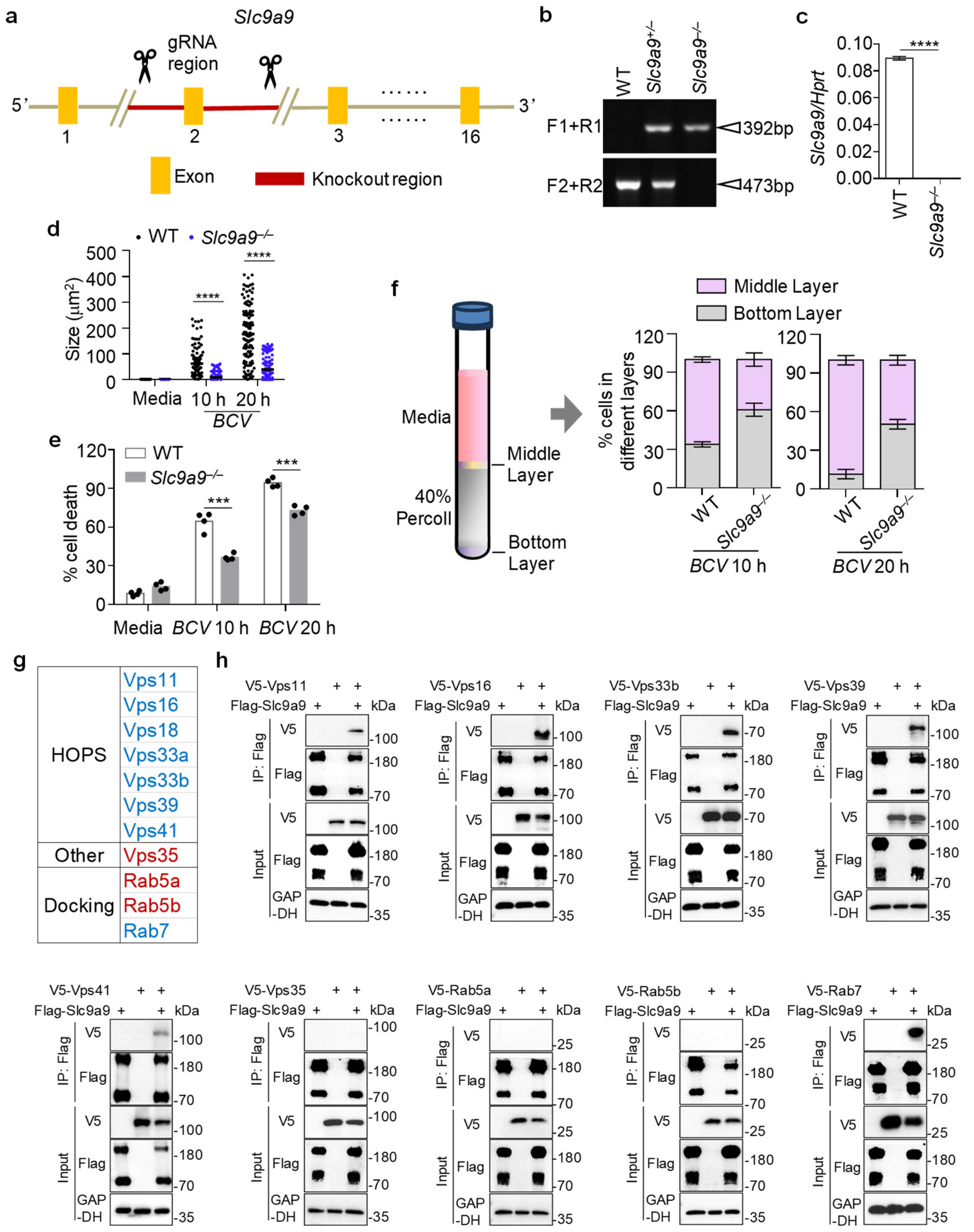

Supplementary Figure S7. The effect of SLC9A9 for *BCV* infection-induced cytoplasmic vacuolization.

a-c Targeting strategy used to generate *Slc9a9*$^{-/-}$ mice (**a**), genotyping of offspring generated from breeding of *Slc9a9* heterozygous mice (**b**), and quantitative RT-PCR analysis of *Slc9a9* in WT and *Slc9a9*$^{-/-}$ BMDMs (**c**) (n = 2 technical replicates; 3 independent experiments).

d Quantification of vacuole size in BMDMs in (Fig. 4f). The largest vacuole per cell was analyzed, and at least 130 cells were quantified for each group.

e LDH analysis of *BCV*-infected WT and *Slc9a9*$^{-/-}$ BMDMs (400 MOI) for the indicated time (n = 4 biologically independent samples).
f Quantification of the level of cytoplasmic vacuolization in *BCV*-infected WT and *Slc9a9*$^{-/-}$ BMDMs (400 MOI) by 40% Percoll gradient separation (n = 3 biologically independent samples).
g,**h** SLC9A9 interacts with HOPS proteins and Rab7. (**g**) The list includes proteins tested for binding to SLC9A9. Blue labels indicate confirmed interactors; red labels indicate proteins that showed no interaction. (**h**) Immunoblot analysis of Flag-SLC9A9 co-IP with V5-Vps11, V5-Vps16, V5-Vps33b, V5-Vps39, V5-Vps41, V5-Vps35, V5-Rab5a, V5-Rab5b, and V5-Rab7 from lysates of HEK293T cells transfected with the indicated plasmids.
Data are from 3 independent experiments (**e, f**) or representative of 3 (**b**-**d**, **h**) independent experiments with similar results. Data represent Mean ± SEM for (**c**-**e**), ***P < 0.001, ****P < 0.0001, by two-sided Student's *t*-test without multiple-comparisons correction.

a

SNAREs	
Stx2	Vamp2
Stx11	Vamp3
Stx12	Vamp4
Stx18	Vamp8
Stxbp1	Sec22b
Stxbp2	Bnip1
Stxbp3	Bet1
Snap29	Bet1L
Snap47	Esyt1
Snapin	Ehd1

b

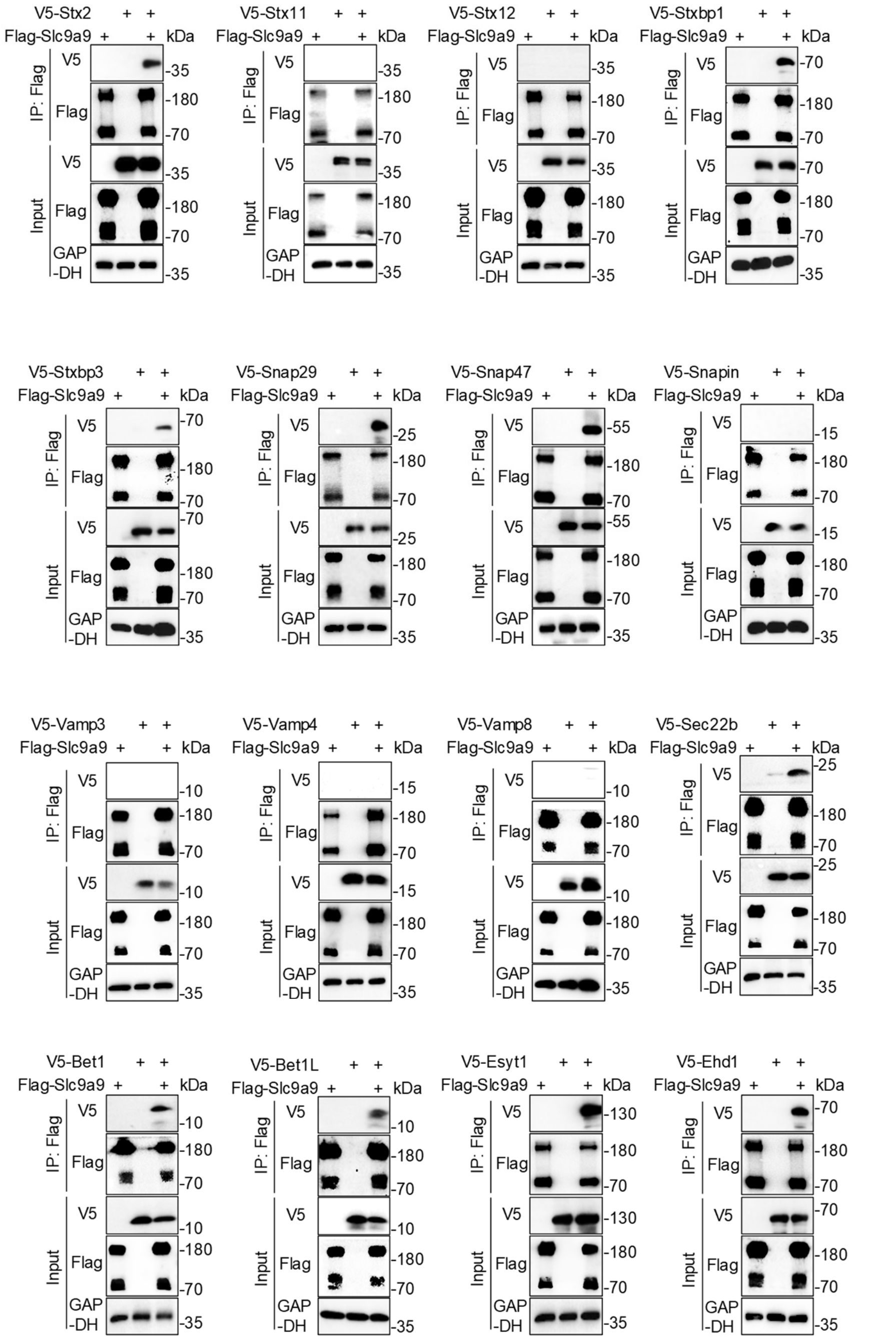

Supplementary Figure S8. SLC9A9 interacts with SNAREs.

a The list includes proteins tested for binding to SLC9A9. Blue labels indicate confirmed interactors; red labels indicate proteins that showed no interaction.

b Immunoblot analysis of Flag-SLC9A9 co-IP with V5-Stx2, V5-Stx11, V5-Stx12, V5-Stxbp1, V5-Stxbp2, V5-Stxbp3, V5-Snap29, V5-Snap47, V5-Snapin, V5-Vamp2, V5-Vamp3, V5-Vamp4, V5-Vamp8, V5-Sec22b, V5-Bnip1, V5-Bet1, V5-Bet1L, V5-Esyt1, and V5-Ehd1 from lysates of HEK293T cells transfected with the indicated plasmids.

Data are representative of 3 independent experiments with similar results.

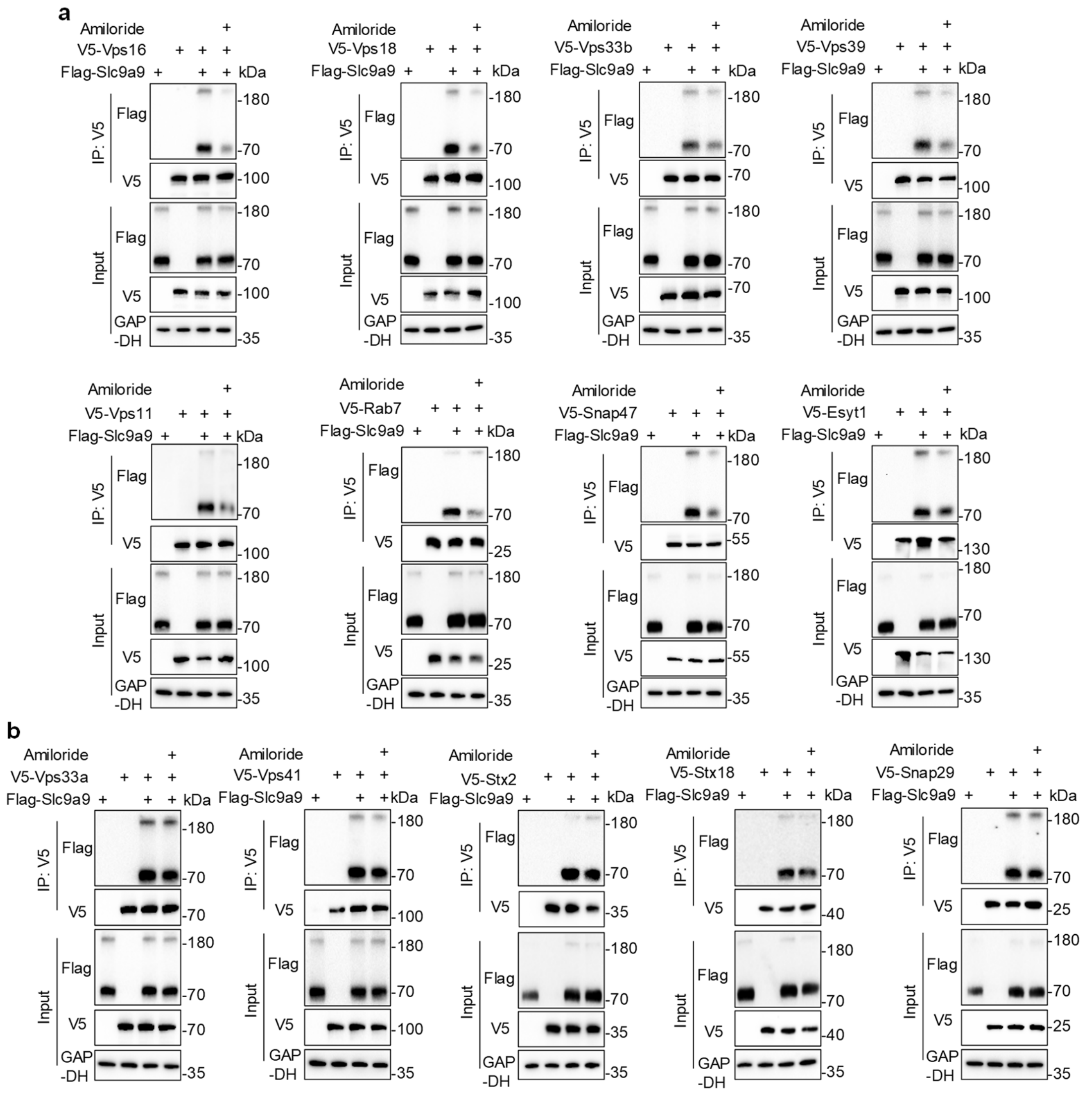

Supplementary Figure S9. Amiloride inhibits SLC9A9 interaction with certain proteins of HOPS and SNAREs.

a Immunoblot analysis of Flag-SLC9A9 co-IP with V5-Vps11, V5-Vps16, V5-Vps18, V5-Vps33b, V5-Vps39, V5-Rab7, V5-Snap47, and V5-Esyt1 from lysates of HEK293T cells transfected with the indicated plasmids in the presence and absence of amiloride hydrochloride (Amiloride, 0.5 μM) treatment.

b Immunoblot analysis of Flag-SLC9A9 co-IP with V5-Vps33a, V5-Vps41, V5-Stx2, V5-Stx18, and V5-Snap29 from lysates of HEK293T cells transfected with the indicated plasmids in the presence and absence of amiloride hydrochloride (Amiloride, 0.5 μM) treatment.

Data are representative of 2 independent experiments with similar results.

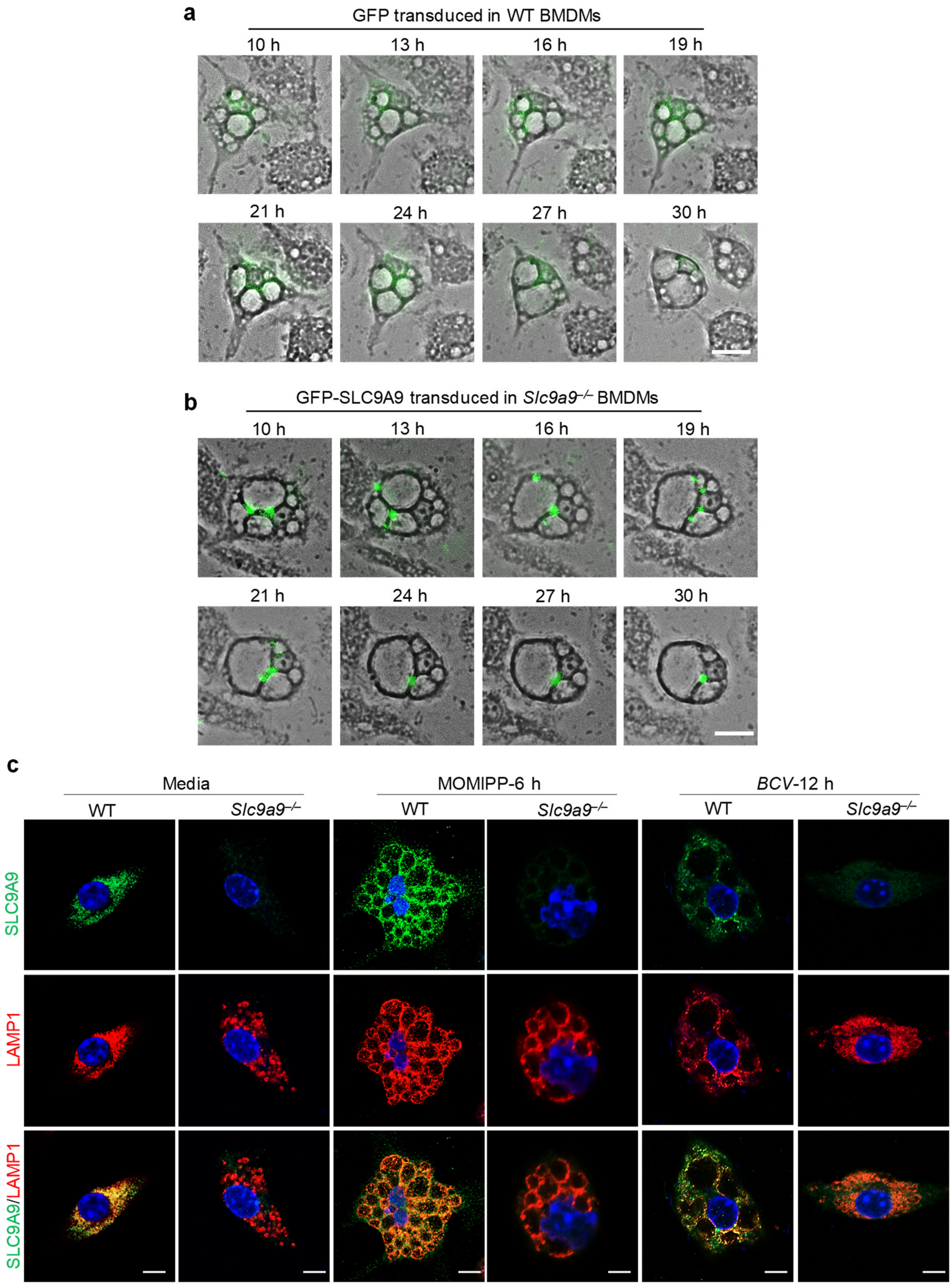

Supplementary Figure S10. SLC9A9 is not required for MOMIPP-induced methuosis.

a Live-cell imaging analysis of the localization of GFP in WT BMDMs transduced with GFP expressing plasmid and infected with *BCV* (400 MOI) for the indicated time. Scale bar, 20 μm.

b Live-cell imaging analysis of the localization of GFP-SLC9A9 in *Slc9a9*$^{-/-}$

BMDMs transduced with GFP fusion with SLC9A9 plasmid and infected with *BCV* (400 MOI) for the indicated time. Scale bar, 20 μm.

c Confocal microscopy analysis of SLC9A9 and LAMP1 in WT and *Slc9a9*$^{-/-}$ BMDMs without treatment (Media), treated with MOMIPP (3 μM) for 6 h, or infected with *BCV* (400 MOI) for 12 h. Scale bars, 10 μm.

Data are representative of 3 independent experiments with similar results.

a

Protein identification	Coverage	Intensity (*BC*)	Intensity (*BCV*)
PorV (DBX24_07555)	3.8	0	44371
Lipocalin (DBX24_06710)	9.6	0	46040
β-barrel (DBX24_07470)	7.7	0	41600
ADSL (DBX24_07765)	3.2	0	10353
OmpA (DBX24_05085)	29.5	3699900	2695000
OmpA (DBX24_02805)	3.2	74052	8371.5
OmpA (DBX24_06715)	7	21730	0
OmpH (DBX24_04640)	14.5	724340	0

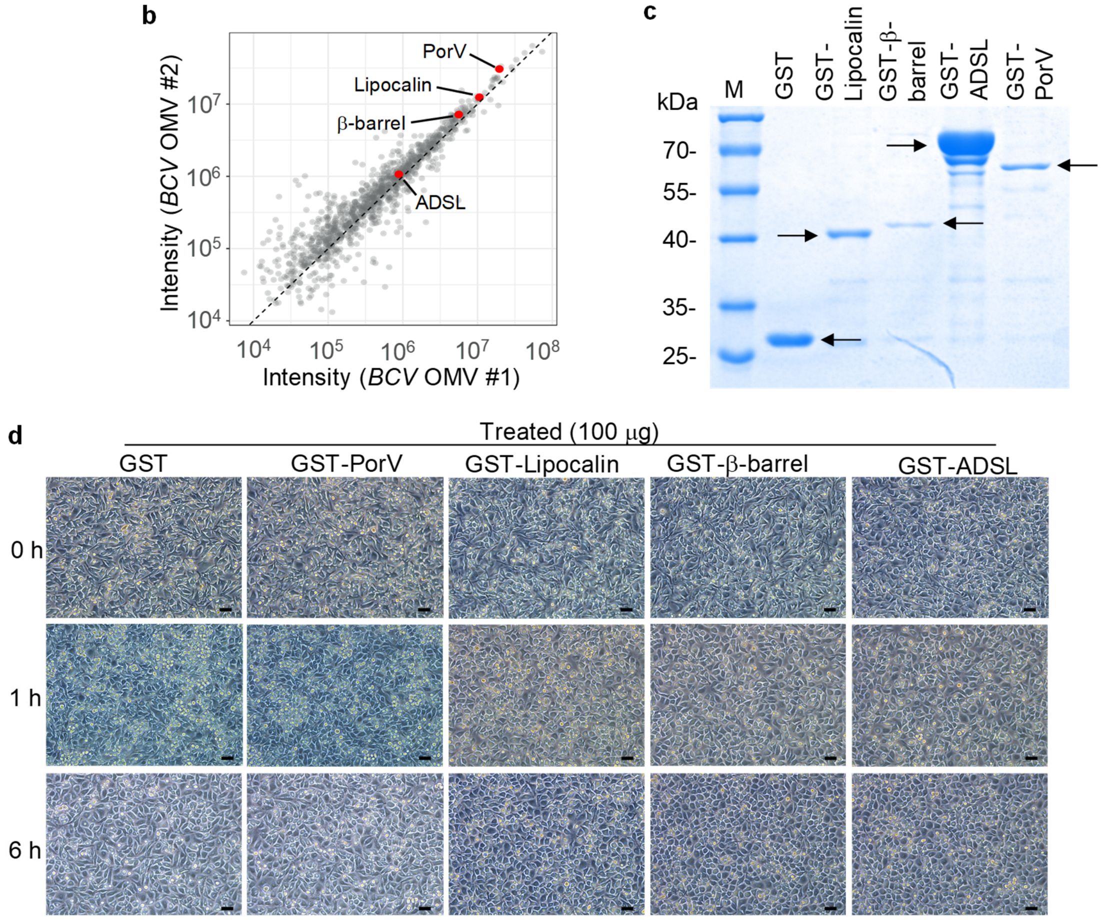

Supplementary Figure S11. Identification and recombinant protein expression of Lipocalin, β-barrel, PorV, and ADSL from *BCV*.

a MS analysis of bacterial proteins in the supernatants from *BCV*- or *BC*-infected (400 MOI) BMDMs for 20 h.

b MS analysis of bacterial proteins in OMVs derived from *BCV*.

c SDS-PAGE of purified recombinant GST-Lipocalin, GST-β-barrel, GST-ADSL, GST-PorV, and GST control proteins, detection was performed by staining with coomassie blue. The arrows indicate the purified proteins.

d Microscopic analysis of WT BMDMs treated with proteins of GST (100 μg), GST-PorV (100 μg), GST-Lipocalin (100 μg), GST-β-barrel (100 μg), and GST-ADSL (100 μg) for the indicated time. Scale bars, 30 μm.

Data are from 2 independent experiments (**b**) or representative of 3 independent experiments with similar results (**c**, **d**).

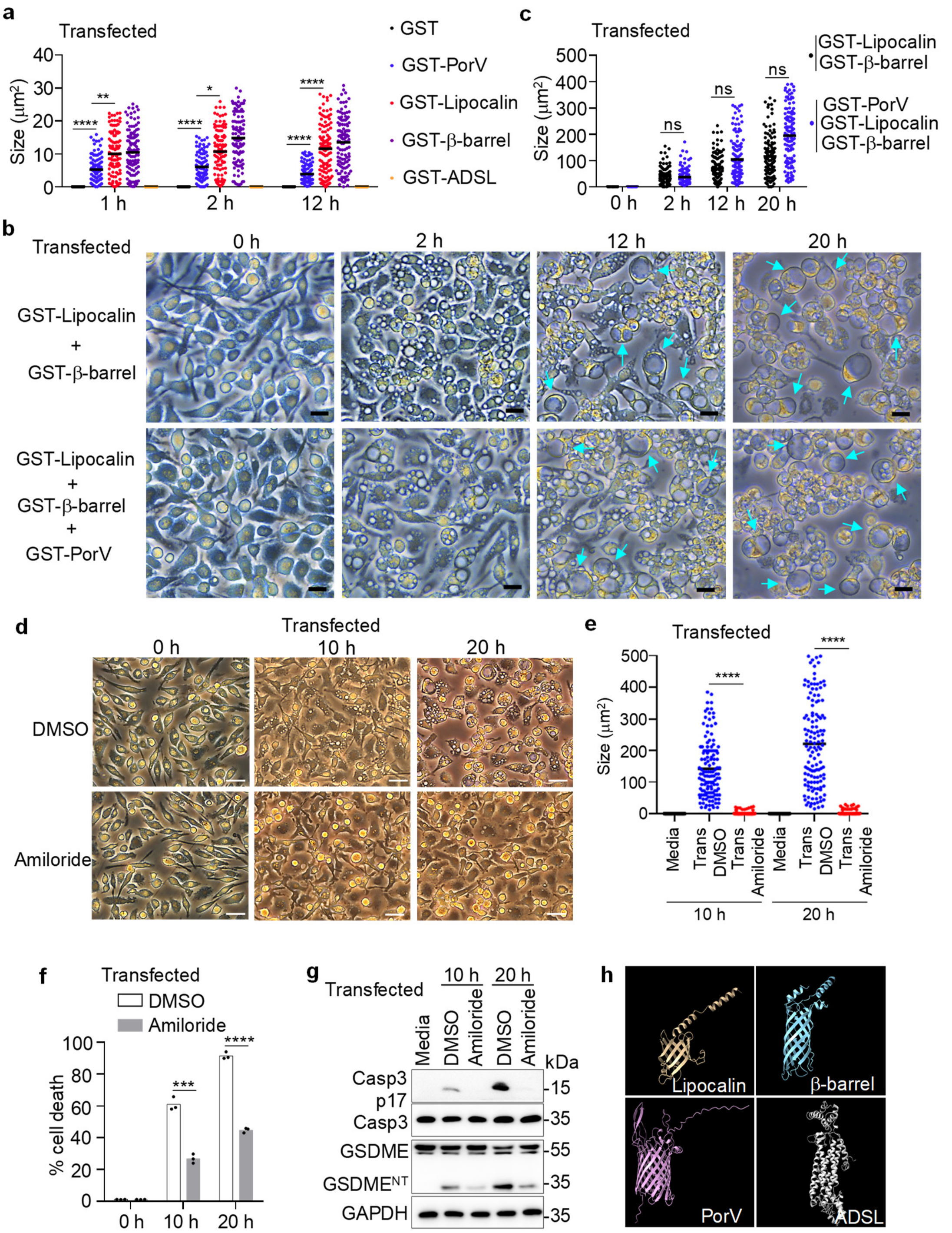

Supplementary Figure S12. Amiloride inhibits cytoplasmic vacuolization cell death induced by transfection of barrel-like proteins.

a Quantification of vacuole size in BMDMs in (Fig. 6a). The largest vacuole per cell was analyzed, and at least 120 cells were quantified for each group.

b Microscopic analysis of WT BMDMs transfected with the combination of GST-Lipocalin (10 µg), GST-β-barrel (10 µg), and GST-PorV (10 µg) proteins for the indicated time. The arrows indicate vacuole-occupied cells. Scale bars, 30 µm.

c Quantification of vacuole size in BMDMs in **b**. The largest vacuole per cell was analyzed, and at least 120 cells were quantified for each group.
d Microscopic analysis of WT BMDMs transfected with the combination of GST-Lipocalin (10 μg), GST-β-barrel (10 μg), and GST-PorV (10 μg) proteins in the presence and absence of amiloride hydrochloride (Amiloride, 0.5 μM) treatment for the indicated time. Scale bars, 30 μm.
e Quantification of vacuole size in BMDMs in **d**. The largest vacuole per cell was analyzed, and at least 120 cells were quantified for each group.
f LDH analysis of WT BMDMs transfected with barrel-like proteins in **d** (*n* = 3 biologically independent samples).
g Immunoblot analysis of caspase-3, cleaved caspase-3 (P17), GSDME, and cleaved GSDME ($GSDME^{NT}$) in WT BMDMs transfected with barrel-like proteins in **d**.
h Predicted protein structure of Lipocalin, β-barrel, PorV, and ADSL from *BCV* by trRosseta.
Data are from 3 independent experiments (**f**) or representative of 3 independent experiments with similar results (**a**-**e**, **g**). Data represent Mean ± SEM for (**a**, **c**, **e**, **f**), *P < 0.05, **P < 0.01, ***P < 0.001, ****P < 0.0001, by two-sided Student's *t*-test without multiple-comparisons correction.

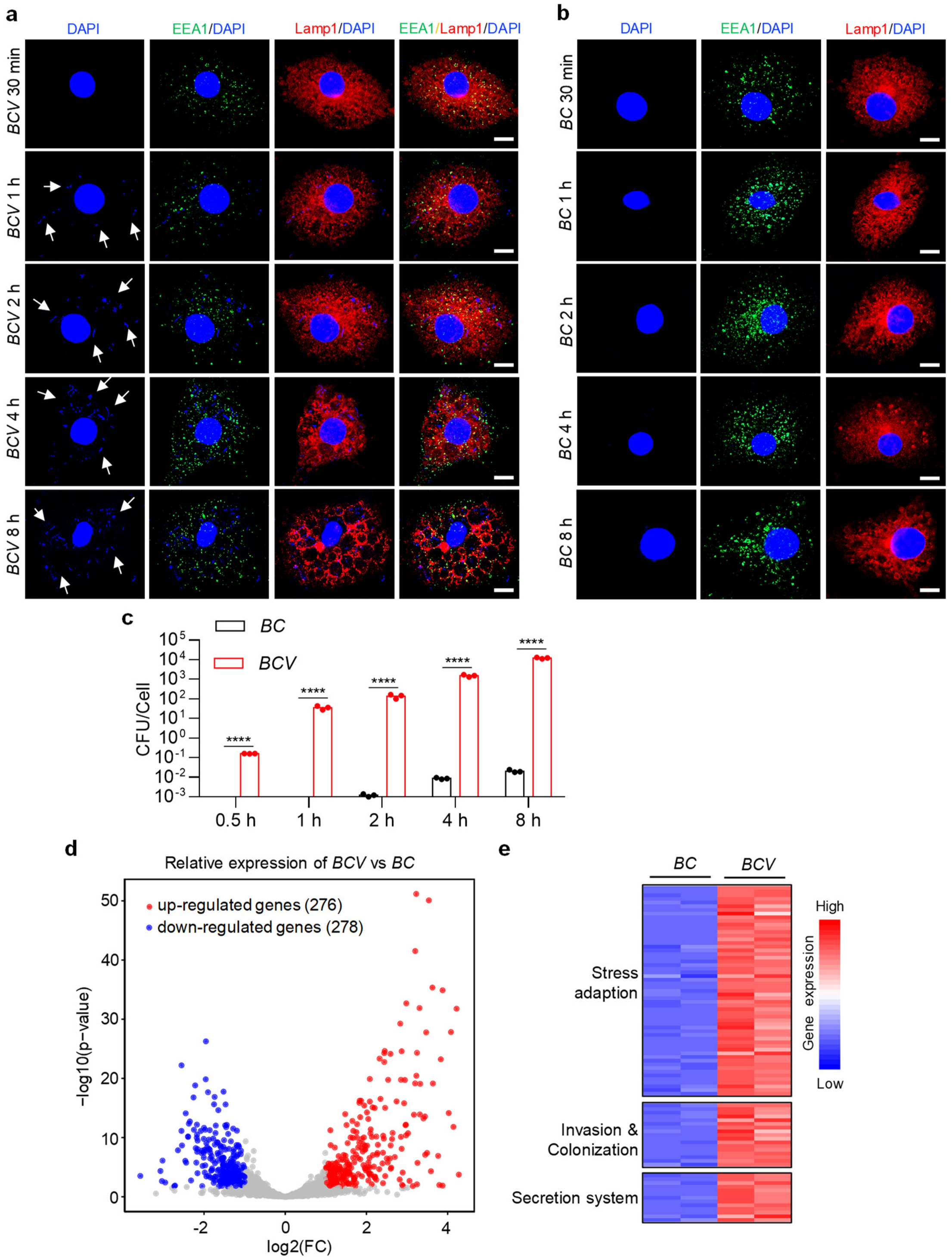

Supplementary Figure S13. Characterization of intracellular *BCV* during infection.

a,**b** Confocal microscopy analysis of intracellular bacteria, EEA1, and LAMP1 in WT BMDMs infected with *BCV* (400 MOI, **a**) and *BC* (400 MOI, **b**) for the indicated time. Arrows indicate the intracellular *BCV*. Scale bars, 10 μm.

c CFU analysis of intracellular *BCV* and *BC* in BMDMs in **a**, **b**. WT BMDMs

were infected with *BC* (400 MOI) and *BCV* (400 MOI) for the indicated time, followed by treating with gentamicin (50 μg/ml) for 1 h. Infected BMDMs were washed, lysed and cultured on Columbia blood agar plates for 96 h for enumeration of intracellular bacteria.

d RNA-seq analysis of the expression of bacterial genes in WT BMDMs infected with *BC* and *BCV* (400 MOI) for 10 h. Both BMDMs and bacteria were collected for RNA sequencing. Volcano plot showing the distribution of upregulated (red) and downregulated (blue) genes at the transcriptional level in *BCV* compared with *BC*.

e Heatmap analysis of genes highly expressed in *BCV* but not in *BC*.

Data are from 2 independent experiments (**d**, **e**) or representative of 3 independent experiments with similar results (**a**-**c**). Data represent Mean ± SEM for (**c**), ****P < 0.0001, by two-sided Student's *t*-test without multiple-comparisons correction.

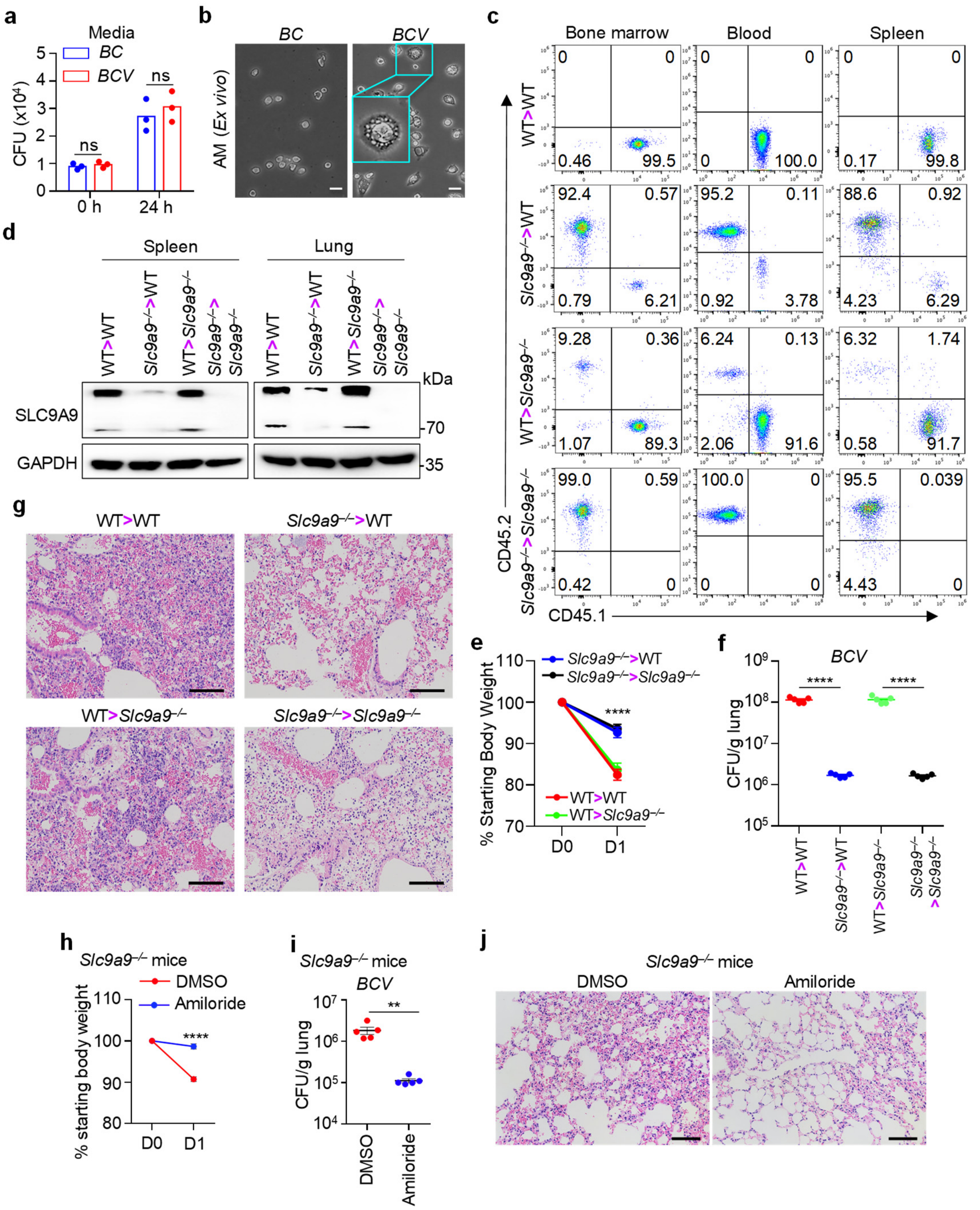

Supplementary Figure S14. Synergic effect of *Slc9a9* deficiency and amiloride administration in host defense against *BCV* infection.

a Growth analysis of *BC* and *BCV* in BMDM culture media. ‘0 h’ indicates the starting point of *BC* and *BCV*, and ‘24 h’ shows the number of *BC* and *BCV* in the media after 24 h of growth (n = 3 biologically independent samples).

b Microscopic analysis of cytoplasmic vacuolization in Alveolar macrophages (AMs) infected with *BC* or *BCV* (400 MOI) for 12 h. Scale bars, 20 µm.

c Representative flow cytometry plots of CD45.1^{+} (from female WT mice) and

$CD45.2^+$ (from female *Slc9a9*$^{-/-}$ mice) cells in bone marrow, peripheral blood and spleens from chimeric mice as indicated. Six to eight h prior to bone marrow transplantation, the recipient mice were irradiated with a dosage of 10 Gy for 10 min. For transplantation, 10 million bone marrow cells were injected into the tail veins of the recipient mice. Six weeks later, bone marrow chimeric mice were intranasally infected with 4.0×10^8 CFU *BCV* (*n* = 5 mice for each group) for further analysis.

d Immunoblot analysis of SLC9A9 in the spleen and lung of chimeric mice as indicated.

e,**f** The body weight change (**e**) and bacterial burden in the lungs of chimeric mice (*n* = 5 mice for each group) as indicated on Day 1 after infection were measured (**f**).

g H&E staining of lung sections from *BCV*-infected chimeric mice in **f** as indicated. Scale bars, 100 μm.

h,**i** *Slc9a9*$^{-/-}$ female mice were intranasally infected with 4.0×10^8 CFU *BCV* in the presence and absence of amiloride administration (*n* = 5 mice for each group), and the body weight change (**h**) and bacterial burden in the lungs on Day 1 after infection were measured (**i**). Amiloride indicates that the mice were intravenously injected with amiloride hydrochloride (10 mg/kg) twice, at 0 and 12 h after *BCV* infection.

j H&E staining of lung sections from DMSO- or amiloride hydrochloride-treated *Slc9a9*$^{-/-}$ mice infected with *BCV* in **i**. Scale bars, 100 μm.

Data are from 2 independent experiments (**e**, **f**) or representative of 3 (**a**-**d**, **g**, **j**) or 2 (**h**, **i**) independent experiments with similar results. Data represent Mean ± SEM for (**a**, **e**, **f**, **h**, **i)**, two-sided Student's *t*-test without multiple-comparisons correction, $^{**}P < 0.01$, $^{****}P < 0.0001$.

cytoplasmic vacuolization cell death

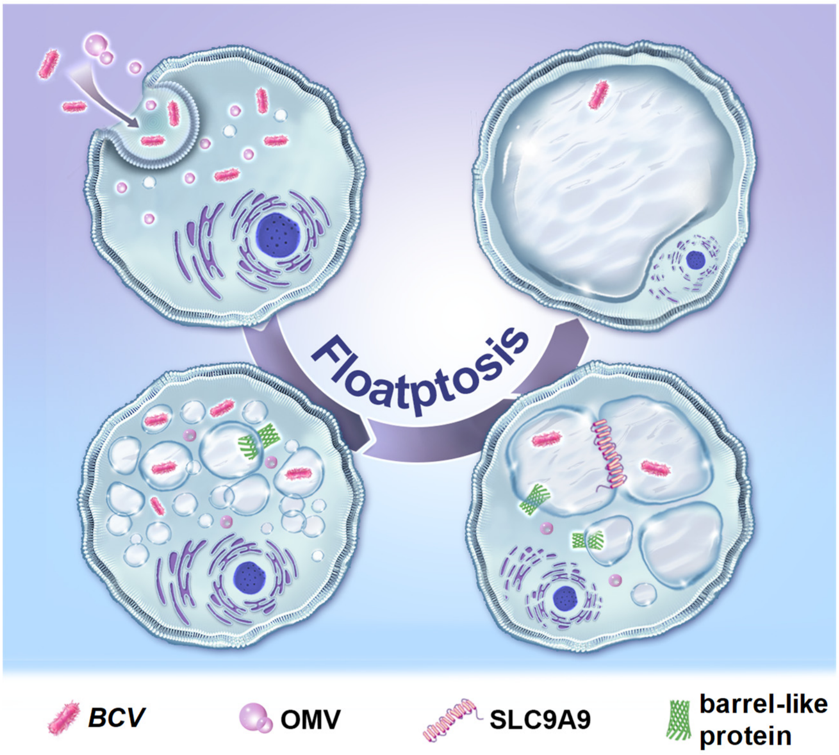

Supplementary Figure S15. A hypothetical model of the mechanisms driving *BCV*-driven cytoplasmic vacuolization cell death. Bacterial pathogens have evolved multiple mechanisms to modulate host cell death. *Bergeyella cardium variant* (*BCV*) triggers unique cytoplasmic vacuolization cell death–fused lysosome-associated termination (floatptosis). The host transmembrane protein SLC9A9, *BCV* outer membrane vesicles (OMVs) and barrel-like proteins play important roles in *BCV*-induced cytoplasmic vacuolization cell death. Figure created by authors and artist Xiaolu Qi.

Supplementary Video S1. Live-cell imaging analysis of the process of vacuolization in WT BMDMs treated with *BCV* (200 MOI) for 40 h. Propidium iodide (PI, red) staining indicates the dead cells with permeable plasma membranes.

Supplementary Table S1. *BC*-unique reads in pan-body pan-disease microbiome sequences.

Supplementary Table S2. RNA expression analysis of *BC* and *BCV*.

Supplementary Table S3. MS analysis of supernatants from *BC*- and *BCV*-infected BMDMs.

Supplementary Table S4. RNA expression analysis of host genes in *BC*- and *BCV*-infected BMDMs.

Supplementary Table S5. MS analysis of OMVs derived from *BCV*.

Supplementary Table S6. RNA expression analysis of bacterial genes in *BC*- and *BCV*-infected BMDMs.

Supplementary Table S7. Oligo sequence.
